# Biomarker and compound-specific isotope records across the Toarcian CIE at the Dormettingen section in SW Germany

**DOI:** 10.1007/s00531-022-02196-z

**Published:** 2022-05-23

**Authors:** Stephen Ajuaba, Reinhard F. Sachsenhofer, Achim Bechtel, Francesca Galasso, Doris Gross, David Misch, Elke Schneebeli-Hermann

**Affiliations:** 1 Lehrstuhl Erdölgeologie, Montanuniversitaet Leoben, Peter-Tunner-Strasse 5, 8700 Leoben, Austria; 2 Paläontologisches Institut und Museum, Universität Zürich, Karl-Schmid-Strasse 4, 8006 Zurich, Switzerland

**Keywords:** Organic geochemistry, Photic zone anoxia, Water column stratification, Bacterial activity, Oceanic anoxic event

## Abstract

The Toarcian oceanic anoxic event (T-OAE) is associated with a prominent negative carbon isotope excursion (CIE; ~ 183 million years (Myr)). About 10-m-thick organic matter-rich sediments accumulated during the T-OAE in the Southwest German Basin (SWGB). Rock–Eval, maceral and biomarker analysis were used to determine variations of environmental conditions across the CIE interval. Carbon isotope records were determined for various *n*-alkanes, pristane and phytane to contribute to the reconstruction of the paleo-environment and to study the factors controlling molecular δ^13^C values. Geochemical redox indicators provide evidence for photic zone anoxia during the Toarcian CIE, which reached its maximum after deposition of the “Unterer Stein” marker horizon. The 2α-methylhopane index suggests enhanced activity of diazotrophic cyanobacteria, which is also supported by nitrogen isotope data. This distinguishes the SWGB from other basins with Toarcian black shale. Oxygen-depleted conditions, albeit with lower intensity continued after the CIE. All investigated compounds replicate the negative CIE, but the magnitudes vary considerably. The largest shift is observed for *n*-C_27_ (9‰) and reflects the combined effect of the global CIE and a major change in organic matter input (termination of terrigenous organic matter input). The shift for short-chain *n*-alkanes, pristane, and phytane, interpreted to reflect marine biomass, varies between 4.5 and 5.0‰. This is the highest value observed so far for any Toarcian section. δ^13^C values of pristane and phytane reach a minimum near the base of the CIE interval and increase upsection. Thus, the maximum negative isotope shift predates the strongest basin restriction by about 450 thousand years (kyr).

## Introduction

The Early Jurassic (Toarcian) is characterized by global climate change (Ruebsam and Schwark 2021 and references therein), a marine extinction event (e.g., Harries and Little 1999; Pálfy and Smith 2000; Dera et al. 2010; Caruthers et al. 2013), and a large (3–8‰) negative carbon isotope excursion (Toarcian CIE) (Hesselbo et al. 2000a, b, 2007). Estimates on the duration of the Toarcian CIE range from 300–500 kyr (Boulila et al. 2014; Boulila and Hinnov 2017) to ~ 620 kyr (Huang and Hesselbo 2014) to 900–1200 ka (Suan et al. 2008, Ruebsam and Al-Hussein 2020).

The Toarcian CIE reflects a perturbation of the global carbon cycle and is potentially associated with the coeval Karoo–Ferrar Large Igneous Province (LIP) (~ 183–182 Ma) (Pálfy et al. 2002; Burgess et al. 2015; Ivanov et al. 2017), but the ultimate trigger for the CIE, which affected the global oceanic and atmospheric carbon reservoirs (e.g., Hesselbo et al. 2000a, b), is still disputed (Silva et al. 2021). Carbon degassing from the Karoo–Ferrar LIP (Pálfy and Smith 2000), generation and release of thermogenic methane during emplacement of the Karoo–Ferrar LIP (e.g., Wignall 2001; Svensen et al. 2007), recycling of isotopically light dissolved inorganic carbon from deeper levels of a stratified water body (e.g., Küspert 1982; Jenkyns 1985; van de Schootbrugge et al. 2005), dissociation of methane hydrate in marine sediments (e.g., Hesselbo et al. 2000a, b; Kemp et al. 2005), increased methane emission from expanding swamps and wetlands (Them et al. 2017), melting of permafrost (Ruebsam et al. 2019; Ruebsam and Schwark 2021), or increased CO_2_ release from the decomposition and oxidation of terrigenous organic matter associated with increased fungal activity (Pienkowski et al. 2016) have been discussed recently. Regardless of the trigger of the carbon release event, high temperatures and an enhancement of the hydrological cycle resulted in strongly (sixfold) increased global weathering rates (Cohen et al. 2004; Kemp et al. 2020).

**Fig. 1:**
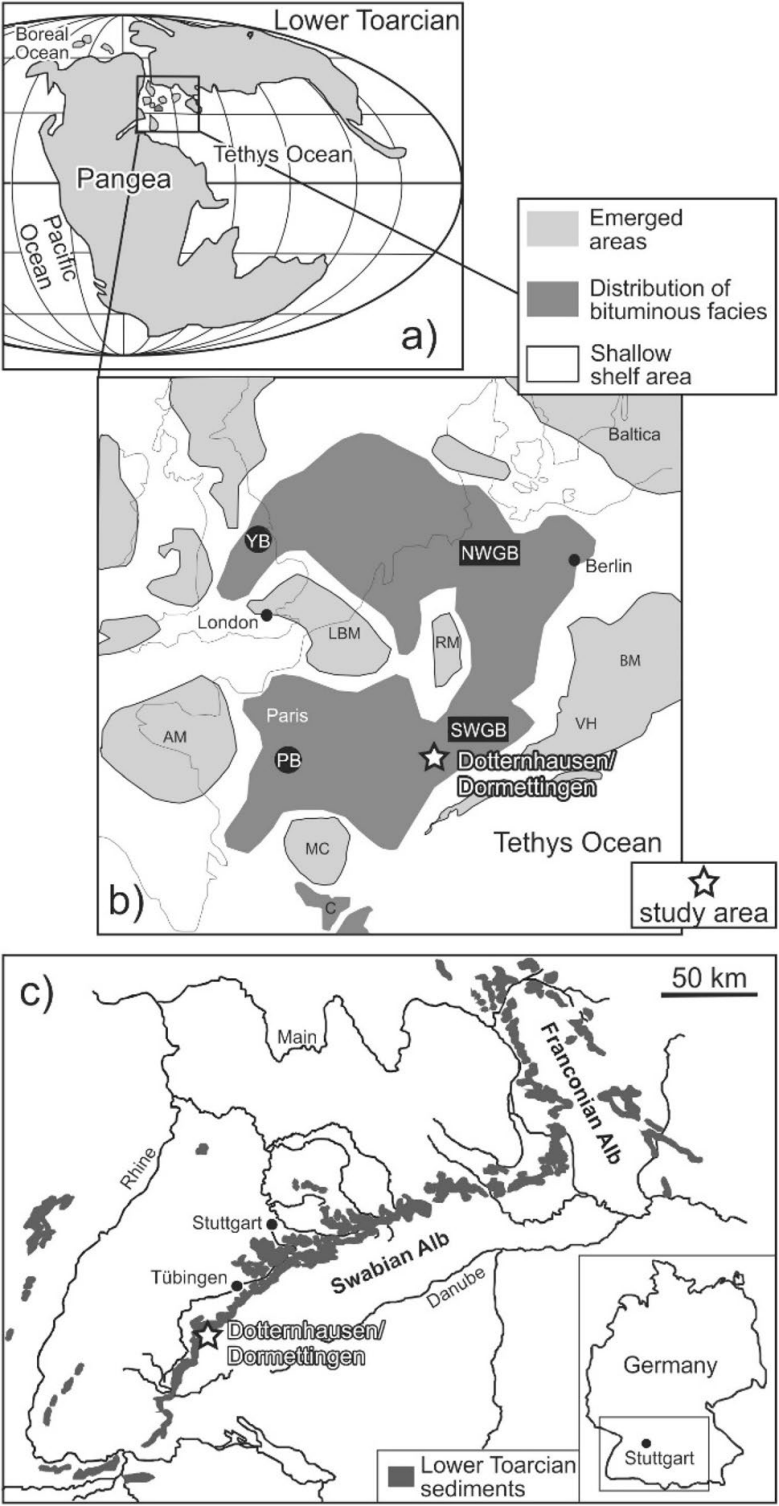
**a** Paleogeographic sketch of the early Toarcian; **b** early Toarcian paleogeography of the epicontinental Central European Basin with the distribution of bituminous black shale across various sub-basins (SWGB: SW German Basin, NWGB: NW German Basin, YB: Yorkshire Basin and PB: Paris Basin); **c** outcrops of the lower Toarcian sediments in southwestern Germany. The white star indicates the study area (Dormettingen). Maps are modified after Röhl et al. (2001), Ziegler (1982) and Galasso et al. (2021)

Black shales with total organic carbon (TOC) contents exceeding 10 wt.% have been deposited during and after the Toarcian CIE in many European basins, characterizing the interval, which is known today as early Toarcian oceanic anoxic event (T-OAE; Jenkyns 1985, 1988, 2010; Neumeister et al. 2015; Silva et al. 2021). Lower Toarcian rocks with very high organic matter contents occur in the Central European Epicontinental Basin System (CEBS; Fig. 1) where they are important petroleum source rocks (e.g., Littke et al. 1991a, b; Cornford 1998; Song et al. 2015). These rocks are known as Posidonia Shale in Germany, Whitby Mudstone in England, or Schistes Carton in France (Jenkyns 1985; Baudin 1995; Röhl and Schmid-Röhl 2005). However, coeval sediment successions from southern Europe (Italy, Portugal, and Spain) and from northern Africa lack organic matter-rich deposits (e.g., de Oliveira et al. 2006; Hesselbo et al. 2007; Rodrigues et al. 2020; Silva et al. 2021).

Detailed carbon isotope records from carbonate minerals and bulk organic matter are available for a great number of sections across the Toarcian CIE (e.g., Röhl et al. 2001; Hesselbo et al. 2007; Kemp et al. 2005, 2020; Hermoso et al. 2012; Suan et al. 2015; Them et al. 2017; for a review see also Ruebsam and Al-Hussein 2020). In contrast, few papers used carbon isotope data of specific organic compounds (e.g., Pedentchouk and Turich 2017), although Schouten et al. (2000), van Breugel et al. (2006), French et al. (2014), Ruebsam et al. (2020) and Xu et al. (2021) indicated their great potential as a proxy for marine and atmospheric carbon isotope ratios. The most complete data set has been published by French et al. (2014). These authors used molecular (biomarker) analysis to reconstruct the depositional environment of the Hawsker Bottoms section in the Cleveland Basin (England) and reported in parallel compound-specific δ^13^C records of short- and long-chained n-alkanes and isoprenoids.

The present paper uses a similar approach and has several goals. The first goal is to reconstruct the of the lower Toarcian rocks in the Southwest German Basin using biomarker data, the second goal is to study differences in carbon isotope trends of bulk organic matter and those of specific molecular fossils (*n*-alkanes, pristane, phytane). Finally, the comprehensive interpretation of biomarker and compound-specific isotope (CSI) data will help to determine the factors controlling CSI patterns and will improve the understanding of the time relation between the development of anoxia and processes, which influence carbon isotopy.

**Fig. 2:**
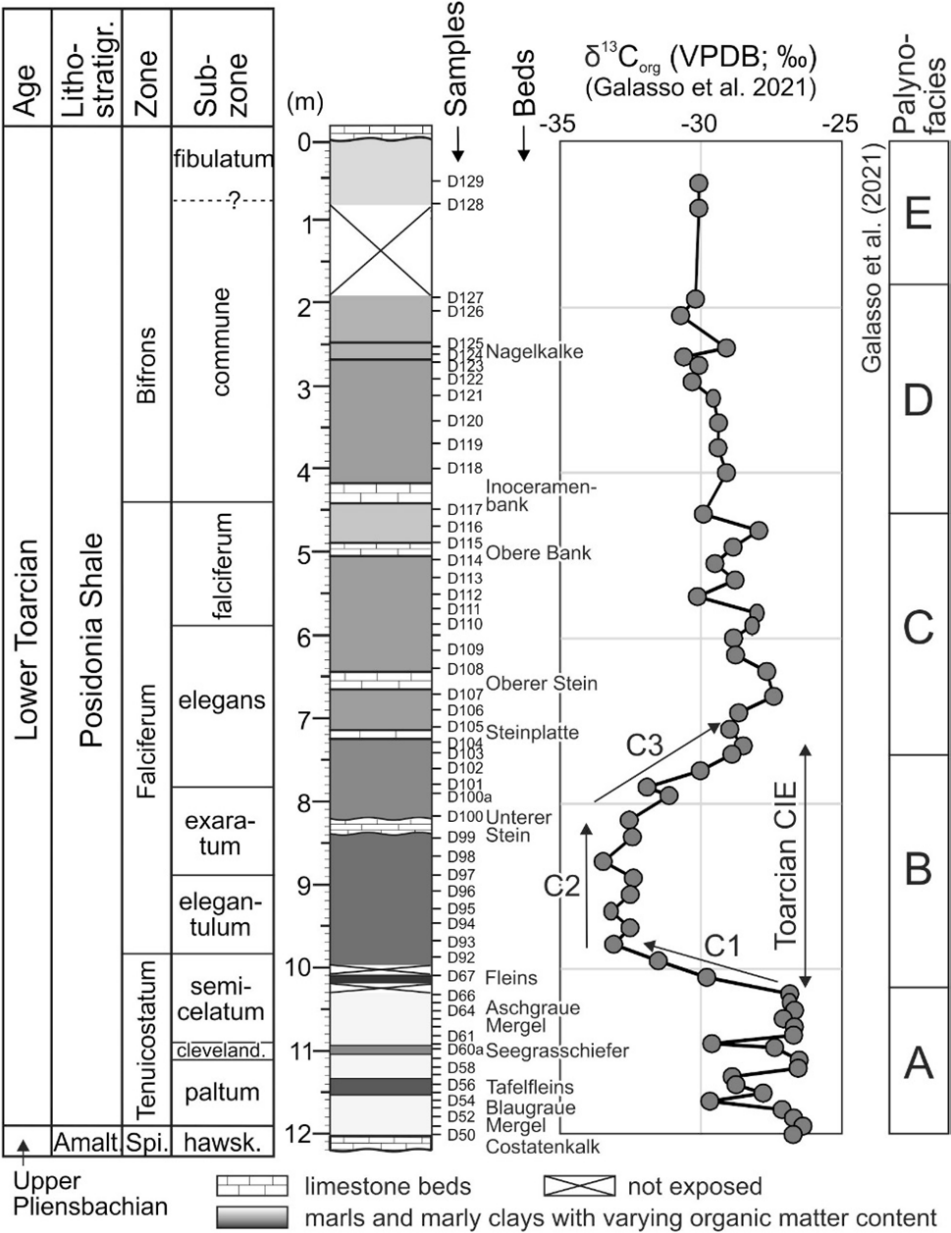
Stratigraphy of the Dormettingen section with ammonite zones and subzones, bulk organic carbon isotope curve, and subdivision into palynofacies intervals (after Galasso et al. 2021). The subdivision of the Toarcian CIE into phases C1–C3 follows Suan et al. (2008). Position of samples investigated by Galasso et al. (2021) (D50–D67; D92–D129) is shown. The identical samples are also used in the present study. (Because of limited space, some samples are unlabeled)

The study is based on sample material from the 12-m-long Dormettingen section, which has been investigated previously regarding bulk organic matter isotopy, palynofacies, and palynology (Galasso et al. 2021; Galasso et al. subm.; Fig. 2; for location see Fig. 1). In addition, Re-Os data are available for this section (van Acken et al. 2019). Another advantage of Dormettingen is its location only 2 km northwest of the Dotternhausen section, which has been studied in great detail using different sedimentological, paleontological, and geochemical techniques (e.g., Riegraf 1985; Schouten et al. 2000; Röhl et al. 2001; Röhl and Schmid-Röhl 2005, van de Schootbrugge et al. 2005; Bour et al. 2007; Mattioli et al. 2008; Suan et al. 2008, 2015; Wang et al. 2020, 2021). This section is no longer accessible, but the presence of marker horizons allows the detailed correlation of data from Dotternhausen to the Dormettingen quarries.

## Geological setting

The epicontinental Central European Basin covered large parts of central and northern Europe during the Jurassic. It widened towards the Tethys Ocean and included several basins that were separated by submarine shoals and island of variable size (Ziegler 1982, 1988) (Fig. 1a). During Toarcian time, a pronounced topography together with climate change and eustatic sea level variations (see Ruebsam and Al-Hussein 2021 for a review) caused stratified water bodies with strongly depleted oxygen contents in a geographically wide area of the CEBS (e.g., Röhl et al. 2001; Schwark and Frimmel 2004; Hermoso et al. 2013; Song et al. 2015, 2017).

The Southwest German Basin, where the study site is located, was located at the southeastern fringes of the Central European Basin System (Fig. 1a). It was connected with the Paris Basin (Hollander et al. 1991; Hermoso et al. 2009, 2014; Song et al. 2014), the Northwest German Basin (e.g., Littke et al. 1991a) and the Cleveland Basin (Hesselbo et al. 2000a, b; Bowden et al. 2006; Kemp et al. 2011; French et al. 2014). The Vindelician High (VH in Fig. 1b) separated the Southwest German Basin from the Tethys Ocean till ongoing transgression caused flooding of the Vindelician High in the Middle/Late Jurassic (Meyer and Schmidt-Kaler 1996).

The Posidonia Shale including the Toarcian CIE and the T-OAE in the Southwest German Basin has been described in great detail (e.g., Schouten et al. 2000; Röhl et al. 2001; Frimmel et al. 2004; Röhl and Schmid-Röhl 2005; van de Schootbrugge et al. 2005; Bour et al. 2007, Schwark and Frimmel 2004; Suan et al. 2008, 2015; Hougård et al. 2021). The following description of the Posidonia Shale in the Southwest German Basin is based on the Dormettingen section (van Acken et al. 2019; Galasso et al. 2021; Fig. 2), which is the focus of the present study.

The Posidonia Shale is underlain by light- to dark-grey bioturbated marls and marly limestones which are assigned to the Pliensbachian Amaltheenton Formation (*Pleuroceras spinatum* Zone). The presence of a diverse benthic fauna implies high oxygen availability (Röhl and Schmid-Röhl 2005). A limestone bed (Costatenkalk), tens of cm thick, marks the uppermost bed of the Amaltheenton Formation (Riegraf 1985; Röhl et al. 2001; Röhl and Schmid-Röhl 2005).

The Posidonia Shale follows above the Costatenkalk without a break in sedimentation (Galasso et al. 2021). The 12-m-thick succession includes the uppermost part of the *Dactylioceras tenuicostatum* ammonite zone, the *Harpoceras falciferum* and *Hildoceras bifrons* zones and their subzones (Fig. 2) (Riegraf 1985; Röhl et al. 2001; Röhl and Schmid-Röhl 2005).

Sediments of the *tenuicostatum* Zone are dominated by light-grey bioturbated marls with benthic fauna (Blaugraue Mergel, Aschgraue Mergel). Two black shale layers (Tafelfleins, Seegrasschiefer), each several centimeters thick, are intercalated between the marls. The Fleins Bed near the top of the *tenuicostatum* Zone signals the onset of Toarcian CIE and continuous black shale deposition, which continued during the *falciferum* and *bifrons* Zones.

Black shales with wavy and lenticular lamination were deposited during the *falciferum* Zone, but lamination becomes less distinct in its upper part (*falciferum* Subzone; van Acken et al. 2019). The black shale succession is marked by the intercalation of five prominent limestone beds which form regional marker horizons. They are labeled from base to top: Unterer Stein (20–30 cm thick), Steinplatte (~ 10 cm thick), Oberer Stein (~ 20 cm thick), Obere Bank (~ 10 cm thick) and Inoceramenbank (~ 10 cm thick) (Fig. 2). The lowermost limestone bed (Unterer Stein; *exaratum* Subzone) is used as lower Toarcian marker horizon in the southern part of the Central European Basin (Riegraf 1982; van de Schootbrugge et al. 2005). The Inoceramenbank marks the boundary between the *falciferum* and *bifrons* Zones. Dark shales in the *bifrons* Zone show indistinct lamination.

Bulk organic carbon isotope data indicate that the Toarcian CIE in the Dormettingen section is located between 10.1 and 7.3 m (uppermost *semicelatum* to middle *elegans* Subzones; Fig. 2) and that the Steinplatte marker bed forms the top of the CIE (Galasso et al. 2021). This is in agreement with the extension of the CIE at Dotternhausen according to Mattioli et al. (2008). However, a slightly higher position (e.g., Suan et al. 2008) seems also possible. Using carbon isotope trends of bulk organic matter, Suan et al. (2008) subdivided the Toarcian CIE at Dotternhausen into three phases: a phase with strongly decreasing δ^13^C values near the *tenuicostatum-falciferum* boundary (C1), a phase of rather constant negative values (C2) and a phase of marked δ^13^C increase (C3) which starts near the top of the Unterer Stein marker bed (Fig. 2). Durations of ~ 150, ~ 450 and 300 kyr have been estimated for C1, C2 and C3, respectively, by these authors. If these estimates are correct, accumulation rates were very low during the Toarcian CIE (~ 3 mm/kyr) (cf. Tyson 2001). The amplitude of the CIE is very high (~ 7‰), but Suan et al. (2015) emphasized that part of the CIE is due to changes in organic matter composition.

Particulate organic matter (POM) data justify the subdivision of the Posidonia Shale into five palynofacies intervals, labeled from base to top A to E (Galasso et al. 2021; Fig. 2). Palynofacies A is documented in POM assemblages from below the Fleins Bed (*tenuicostatum* Zone) and is characterized by high amounts of terrigenous phytoclasts sporomorphs and dinoflagellate cysts. Palynofacies B coincides with the Toarcian CIE. Its assemblages are characterized by high amounts of amorphous organic matter and the lack of dinoflagellate cysts. Dinoflagellate cysts reappear in palynofacies C, which includes the remaining part of the *falciferum* Zone. Palynofacies D and E occur within the *bifrons* Zone. They are marked by relatively higher (D) and lower (E) contributions of terrigenous phytoclasts and sporomorphs (Galasso et al. 2021).

A Re/Os age from the Dormettingen section (183.0 ± 2.0 Ma) agrees well with previous estimates of the age of the Toarcian CIE (van Acken et al. 2019). Low initial ^187^Os/^188^Os_i_ ratio (0.377 ± 0.065) is consistent with a significant influx of mantle-derived material into the Toarcian ocean from weathering of Karoo–Ferrar LIP basalts (van Acken et al. 2019).

## Samples and analytical methods

The present paper is based on rock samples which have been collected for palynological and carbon isotope studies (Galasso et al. 2021). The stratigraphic position of the samples within the Dormettingen section (D50–D67; D92–D129) is shown in Fig. 2. Samples D50–D127 were collected in October 2018 from freshly mined areas of the Dormettingen quarry, while the uppermost samples (D128–D129) are from older parts of the quarry, which was opened in 2008. The sample size is small, between 0.5 and 2.0 cm^3^, to minimize time averaging effects. The remains of three samples (D92, D95; D123) were too small and did not allow geochemical investigations. Therefore, a total of 56 samples are included in the present study. Sample D99 represents the Unterer Stein, a limestone marker bed in the Toarcian CIE. As both the study of Galasso et al. (2021) and the present study focus on organic material, other limestone beds within the Posidonia Shale were not sampled.

Rock powders from all samples were analyzed for total carbon (TC; %), total sulfur (TS; %) and total organic carbon (TOC; %) using an Eltra Helios CS elemental analyzer. TOC contents were measured on samples pre-treated with concentrated phosphoric acid. TOC and TC were used to calculate the equivalent calcite content (calcite_equiv._ = 8.334 × [TC − TOC]).

Rock–Eval parameters *S*_1_ and *S*_2_ (mgHC/g rock) and *T*_max_ (°C) were determined using a “Rock–Eval 6” instrument. *S*_1_ and *S*_2_ are the amounts of hydrocarbons volatized at 300 °C and during gradual heating from 300 to 650 °C, respectively. *T*_max_ is a maturity parameter and corresponds to the temperature at which a maximum of *S*_2_ hydrocarbons is formed. *S*_1_ and *S*_2_ were used to calculate the hydrogen index (HI = 100 × *S*_2_/TOC) and the production index (PI = *S*_1_/[*S*_1_ + *S*_2_]) (Espitalié et al. 1977).

Based on the bulk geochemical data and their stratigraphic distribution, 24 samples were selected for organic petrography, biomarker, and isotope investigations. Semi-quantitative maceral analysis was performed on polished blocks using white and fluorescence light, a 50 × oil immersion objective, and a Leica DM 4P microscope. Depending on TOC contents, 1000 to 1500 points were counted in a single scan and standardized to 100% organic matter. This maceral analysis was performed in accordance with the ICCP System (ICCP 1998, 2001; Pickel et al. 2017). Vitrinite reflectance measurements were performed on two samples (25–50 measurements per sample) using the same microscope and an yttrium–aluminum–garnet standard (Rr: 0.899%).

For biomarker analysis, 5–10 g of powdered rock samples was extracted for ~ 1 h using a Dionex ASE 350 Accelerated Solvent Extractor with dichloromethane as solvent at 75 °C and 100 bar. The extraction solvent was evaporated to ~ 0.5 ml total solution using a Zymark TurboVap 500 closed cell concentrator. Afterwards, asphaltenes were precipitated from a hexane:dichloromethane solution (80:1 according to volume) and separated by centrifugation.

The hexane-soluble fractions were split into NSO compounds and saturated and aromatic hydrocarbons using the medium pressure liquid chromatography (MPLC) device of Köhnen-Willsch (Radke et al. 1980).

*n*-Alkanes and isoprenoids within the saturated hydrocarbon fractions were analyzed using a gas chromatograph (Trace GC-Ultra) with a flame ionization detector (GC-FID). The gas chromatograph was equipped with a 50 m HP-PONA capillary column (inner diameter [i.d.] 0.20 mm, 0.50 µm film thickness). After sample injection (2 µl at 270 °C), the oven temperature was increased from 70 to 310 °C and held constant for 35 min.

Biomarker molecules in the saturated and aromatic hydrocarbon fractions were analyzed by gas chromatography–mass spectrometry (GC–MS) using a Thermo Scientific Trace GC-Ultra equipped with a Triplus 100 liquid auto-sampler and interfaced to a ThermoFisher ISQ single quadrupole mass spectrometer. The spectrometer was operated in the EI (electron ionization) mode scanning from *m*/*z* 50 to *m*/*z* 650 (0.7 s total scan time). Prior to analysis, proportionate amounts of internal standards (squalane for aliphatics; 1,1´-binaphthyl for aromatics) were added to each sample. Measurements were performed with a 60 m DB-5MS fused capillary column (i.d. 0.25 mm; 0.25 μm film thickness). The GC oven temperature was initially programmed from 40 °C (held for 2 min) and ramped to 310 °C with 4 °C/min, followed by an isothermal period of 40 min. Sample injection was done in a split mode (split ratio 20) at 260 °C using helium as carrier gas. The aliphatic fractions of selected samples were additionally analyzed with a Trace™ 1300 GC (ThermoFisher) equipped with a 60 m TG-5MS fused silica capillary column (i.e., 0.25 mm; 0.25 µm film thickness) coupled to a ThermoFisher TSQ9000 triple quadrupole GC–MS/MS. The oven was programmed to hold 40 °C for 2 min and then heated to 310 °C with 4 °C/min, followed by an isothermal period of 40 min. The sample was injected splitless with an injector temperature of 310 °C. For the detection of methylsteranes isomers, a selected reaction monitoring process (SRM) was used. Different parent-to-daughter ion transitions (*m*/*z* 386–231, 400–231, 414–231, 414–98) were monitored to obtain C_28_ to C_30_ methylsteranes and dinosteranes.

Data were processed with Xcalibur and Chromeleon data system. Individual compound identification was based on their respective retention time within the mass spectra or total ion current (TIC) chromatogram and by comparing the mass spectra with published data. In the case of mass chromatograms, response factors were used to account for corrections of the fragment ions used for quantification of the total ion abundance while for TIC chromatograms, absolute concentrations were calculated for different saturated and aromatic compounds in relation to their internal standards per sample.

A semi-quantitative calculation of the target compounds was conducted based on results of total ion current (TIC) chromatograms. Thereby, methylsteranes which could be detected in TIC (total ions as well as *m*/*z* 231) and for which a calculation of the amount was possible were used for further calculations. Areas of specific compounds were taken then from the SRM mode and were set into relation to the TIC. With this relation, a proper estimation of the amount of methylsteranes was possible.

The *n*-alkanes were separated from branched/cyclic hydrocarbons by an improved 5 Å molecular sieve method (Grice et al. 2008) for the analysis of stable carbon isotope ratios on individual *n*-alkanes and isoprenoids. Compound-specific carbon isotope measurements of the *n*-alkanes and isoprenoids were performed using a Trace GC-Ultra gas chromatograph attached to the ThermoFisher Delta-V isotope ratio mass spectrometer (IRMS) via a combustion and high temperature reduction interface (GC Isolink, ThermoFisher). The GC column is as described above, while the oven temperature was programmed to 70 °C for 2 min followed by a 4 °C/min increment to 300 °C and held for 15 min. For calibration, a CO_2_ standard gas was injected before and after each analysis. Each sample was analyzed in duplicate. The mean isotope composition is reported in the δ notation in permil (‰) relative to the V-PDB standard. Analytical reproducibility of the total procedure is in the range of 0.1 to 0.3‰.

## Results

### Bulk geochemistry

**Fig. 3:**
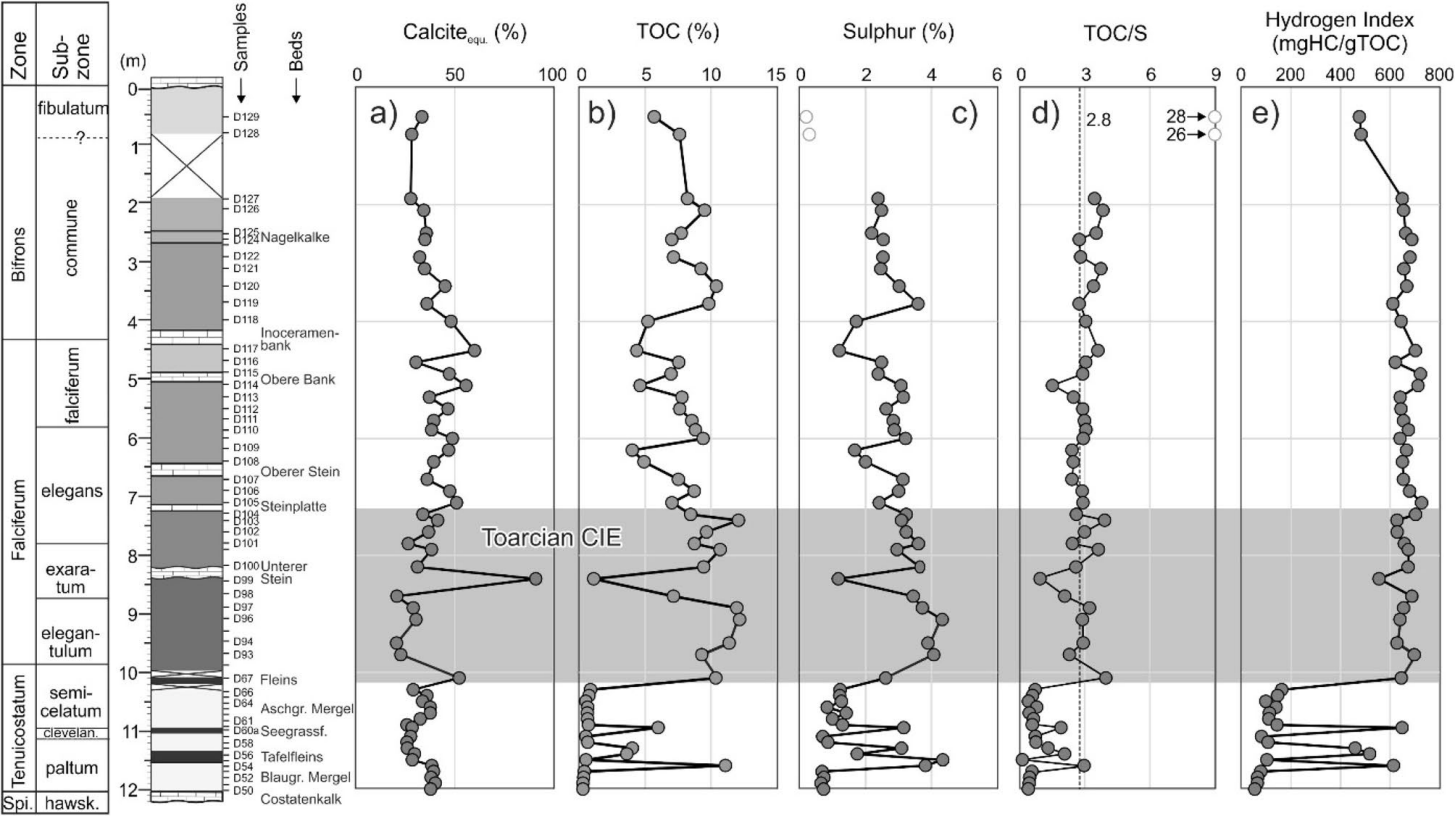
Stratigraphic profile across the Dormettingen section (after Galasso et al. 2021) with calcite equivalent percentages, total organic carbon (TOC) and sulfur (S) contents, TOC/S ratios, and hydrogen index (HI). Sulfur contents and TOC/S ratios of samples D128/129 are probably influenced by weathering and, therefore, shown by open symbols

Inorganic and organic bulk parameters of the Dormettingen section are shown in Fig. 3. Calcite equivalent percentages below the Toarcian CIE range from 25 to 41 wt.%. Within the CIE carbonate contents are relatively low (20–40 wt.%), but high in the limestone marker bed (Unterer Stein: 91 wt.%). Calcite contents of marls above the CIE range from 27 to 60 wt.%. Limestone beds above the CIE have not been investigated, as the focus of the present study lies on the organic matter-rich rocks.

TOC contents in marls below the Toarcian CIE are typically low (0.2–0.9 wt.%). Only sediments related to the Tafelfleins and Seegrasschiefer contain high amounts of organic matter (max. 11.1 wt.%). Apart from the limestone marker (Unterer Stein: 1.1 wt.% TOC), TOC contents in the T-OAE range from 7.1 to 12.2 wt.%. TOC contents remain high above the CIE (4.0–10.4 wt.%).

Sulfur contents range from 0.6 to 4.4 wt.% and show a similar vertical trend than TOC contents. Only the two uppermost samples (*fibulatum* Subzone) contain very low amounts of sulfur (0.2–0.3 wt.%), probably due to weathering. TOC/S ratios below the Toarcian CIE interval are typically below 1, whereas higher ratios are observed in the organic-rich layers. Within the CIE interval TOC/S ratios vary between 2.0 and 4.0. Lower ratios are restricted to the limestone bed (Unterer Stein). Above the CIE interval TOC/S ratios range from 1.5 to 3.9. Very high ratios (~ 30) in the uppermost samples probably reflect weathering (cf Ruebsam et al. 2018).

Hydrogen index (HI) values of low-TOC samples below the Toarcian CIE increase upwards from 53 to 161 mgHC/gTOC. HI of the black shale layers varies between 459 and 649 mgHC/gTOC. Similar values are observed within the CIE (556–699 mgHC/gTOC) and above the CIE (612–728 mgHC/gTOC). HI values in the *fibulatum* Subzone (~ 480 mgHC/gTOC) may be reduced by weathering.

*T*_max_ values vary between 422 and 429 °C (average 427 °C) and show a subtle upward increasing trend (Table 1). The PI ranges from 0.01 to 0.09 (average 0.04).

**Table 1:** Bulk geochemical parameters for Toarcian sediments from the Dormettingen section

Sample ID	Depth [m]	TOC [wt%]	Sulfur [wt%]	Calcite [wt%]	S1	S2	HI [mg HC/g TOC]	PI [–]	*T*_max_ [°C]
					[mg HC/g rock]				
D129	0.5	5.69	0.20	33.4	0.29	27.11	476	0.01	426
D128	0.8	7.62	0.29	28.3	0.48	36.81	483	0.01	422
D127	1.9	8.21	2.38	27.8	1.63	53.39	650	0.03	427
D126	2.1	9.52	2.48	34.3	2.50	62.36	655	0.04	428
D125	2.49	7.74	2.18	35.6	1.60	51.35	663	0.03	428
D124	2.6	7.02	2.54	34.9	1.58	48.39	689	0.03	428
D122	2.9	7.16	2.53	32.2	1.78	48.74	681	0.04	429
D121	3.1	9.26	2.46	34.8	2.58	60.74	656	0.04	429
D120	3.4	10.42	3.02	45.1	3.70	69.55	668	0.05	429
D119	3.7	9.83	3.59	35.9	2.86	60.22	612	0.05	429
D118	4	5.22	1.72	48.1	1.13	33.69	645	0.03	429
D117	4.5	4.37	1.20	60.0	1.10	30.75	703	0.03	429
D116	4.7	7.56	2.49	30.4	1.85	47.02	622	0.04	428
D115	4.9	6.97	2.39	47.3	2.24	50.42	723	0.04	428
D114	5.1	4.62	3.07	55.8	1.19	32.95	713	0.03	427
D113	5.3	7.80	3.15	37.1	2.18	50.07	642	0.04	429
D112	5.5	7.63	2.63	46.5	2.25	49.15	644	0.04	429
D111	5.7	8.54	2.84	39.4	2.63	55.98	655	0.04	428
D110	5.85	8.82	2.87	38.3	2.75	59.55	675	0.04	428
D109a	6	9.40	3.21	48.8	3.42	60.27	641	0.05	428
D109	6.2	4.02	1.67	46.9	0.86	26.87	668	0.03	427
D108	6.4	4.92	2.00	39.4	1.05	32.02	650	0.03	428
D107	6.7	7.53	3.14	36.0	2.11	49.31	655	0.04	427
D106	6.9	8.73	3.00	47.4	3.01	59.35	680	0.05	428
D105	7.1	7.04	2.42	50.9	2.44	51.30	728	0.05	428
D104	7.3	8.47	3.23	33.9	2.42	59.59	704	0.04	427
D103	7.4	12.09	3.09	41.4	4.14	76.04	629	0.05	428
D102	7.6	9.67	3.23	36.8	3.15	60.84	629	0.05	427
D101	7.8	8.75	3.61	26.5	2.44	57.62	658	0.04	429
D101a	7.9	10.70	2.95	38.3	4.48	72.18	674	0.06	426
D100	8.2	9.45	3.65	31.2	3.54	63.64	673	0.05	427
D99	8.4	1.10	1.17	91.0	0.49	6.11	556	0.07	424
D98	8.7	7.16	3.44	20.7	2.33	49.30	689	0.05	426
D97	8.9	11.97	3.73	29.0	4.83	78.45	655	0.06	427
D96	9.1	12.18	4.33	30.4	5.00	78.03	640	0.06	427
D94	9.5	11.38	3.89	20.6	4.19	71.53	628	0.06	427
D93	9.7	9.30	4.07	22.7	3.65	65.02	699	0.05	425
D67	10.1	10.36	2.61	52.2	3.94	66.98	646	0.06	426
D66	10.3	0.85	1.23	29.0	0.04	1.38	162	0.03	428
D65	10.4	0.75	1.22	35.9	0.04	1.08	145	0.04	426
D64	10.5	0.50	1.26	33.7	0.03	0.49	98	0.05	424
D63	10.6	0.65	0.83	37.6	0.04	0.90	139	0.04	427
D62	10.7	0.61	1.42	37.7	0.04	0.69	111	0.05	425
D61	10.8	0.63	1.00	32.6	0.03	0.69	110	0.04	426
D60	10.9	0.70	1.30	25.8	0.04	1.00	143	0.03	426
D60a	10.95	5.99	3.16	28.4	1.25	38.88	649	0.03	427
D59	11.1	0.50	0.70	27.6	0.03	0.40	80	0.07	425
D58	11.2	0.64	0.86	25.8	0.04	0.68	107	0.05	424
D57	11.3	4.03	3.09	26.0	0.48	18.51	459	0.03	425
D56	11.4	3.61	1.75	29.7	0.42	18.70	518	0.02	425
D55	11.5	0.51	4.34	28.5	0.03	0.53	102	0.05	423
D54	11.6	11.10	3.82	38.4	3.06	68.16	614	0.04	425
D53	11.7	0.38	0.68	39.3	0.02	0.29	77	0.06	427
D52	11.8	0.34	0.74	38.0	0.02	0.22	64	0.07	425
D51	11.9	0.28	0.65	40.2	0.02	0.18	63	0.08	426
D50	12	0.28	0.73	37.8	0.02	0.15	53	0.09	425

### Organic petrography

**Fig. 4:**
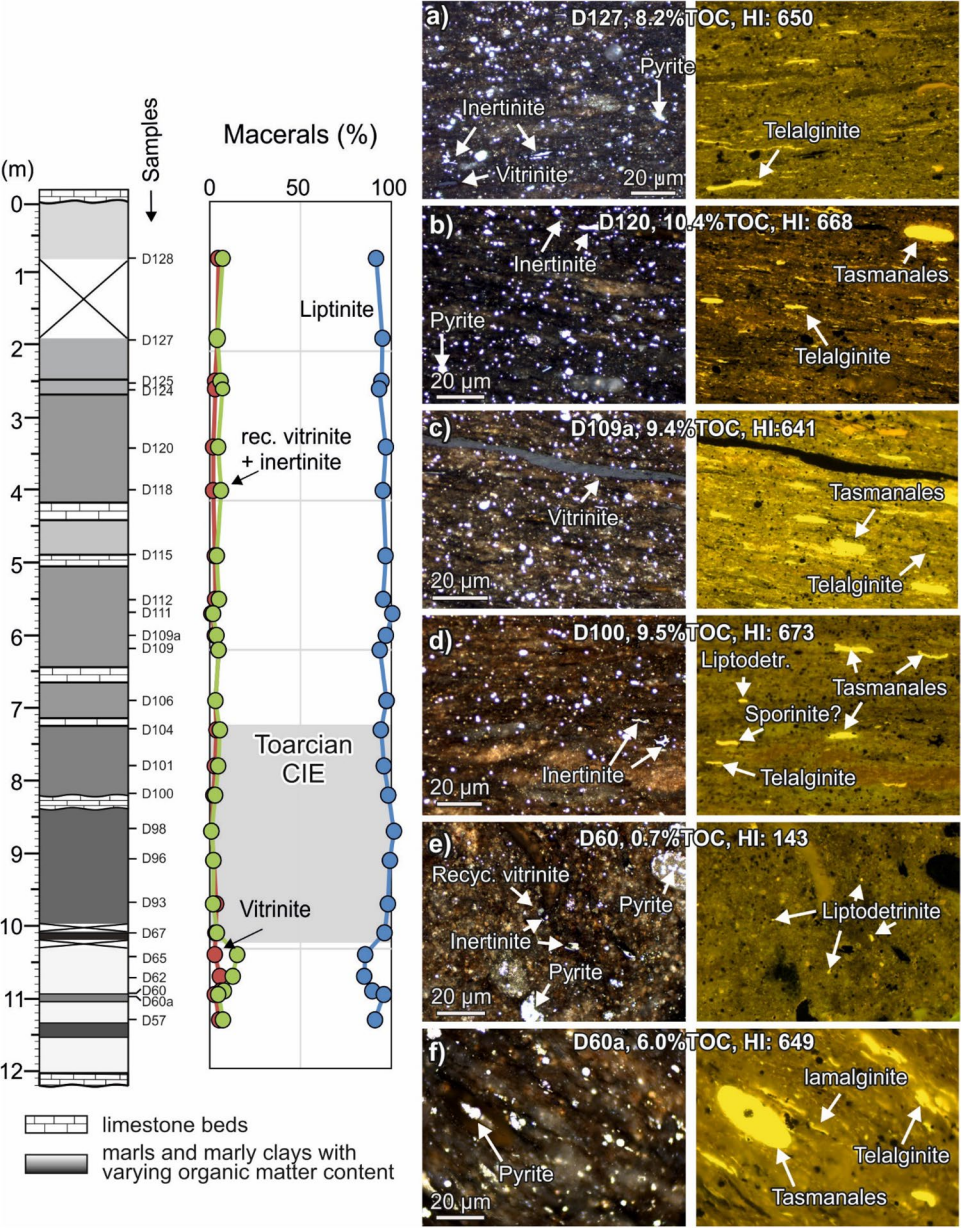
Maceral composition of Dormettingen samples with micro-photomicrographs of selected samples (left: white light; right: fluorescence mode)

Semi-quantitative maceral analysis shows that the organic matter of the Toarcian shales at Dormettingen is dominated by liptinite macerals (Table 2; Fig. 4), particularly alginite (50–81 vol.%) and liptodeterinite (14–41 vol.%). Telalginite dominates over lamalginite in samples below the CIE and in the upper part of the CIE (D98, D100, D101), while lamalginite prevails in most of the remaining samples. Telalginite includes Tasmanales algae beside of significantly smaller telalginite macerals (e.g., Fig. 4b, f). Tasmanales algae are especially abundant in sample D101 from the upper part of the CIE. Liptodeterinite (e.g., Fig. 4d) is probably also derived from algal material and occurs in high quantities (~ 40 vol%) in sample D57 (Tafelfleins) and in samples D98 and D100 from the Toarcian CIE interval. Sporinite is generally very rare and is observed in quantities exceeding 1 vol.% only in sediments above the CIE (Table 2).

**Table 2:** Maceral percentages of Toarcian sediments from the Dormettingen section

Sample ID	Depth [m]	Vitrinite [vol%]	Inertinite (+ recycled vitrinite) [vol%]	Sporinite [vol%]	Tasmanales telalginite [vol%]	Undefined telalginite [vol%]	Lamalginite [vol%]	Liptodetrinite [vol%]
D128	0.8	4	6	1	1	17	55	16
D127	1.9	3	3	0	4	19	39	32
D125	2.49	2	5	0	2	29	36	25
D124	2.6	2	6	0	5	26	35	25
D120	3.4	1	4	1	5	32	39	19
D118	4	1	5	2	9	21	35	28
D115	4.9	2	3	1	2	18	43	32
D112	5.5	2	4	1	4	30	31	29
D111	5.7	0	1	1	1	22	44	32
D109a	6	2	3	1	8	27	46	14
D109	6.2	4	4	0	0	17	44	31
D106	6.9	2	2	0	2	19	45	30
D104	7.3	3	5	0	3	21	47	23
D101	7.8	2	4	0	20	39	4	31
D100	8.2	1	2	0	2	30	24	41
D98	8.7	0	0	0	1	36	22	41
D96	9.1	1	1	0	0	24	41	33
D93	9.7	3	1	0	0	22	42	32
D67	10.1	2	3	0	11	24	35	25
D65	10.4	2	14	0	2	56	5	21
D62	10.7	5	12	0	2	49	12	21
D60	10.9	5	7	0	3	42	19	24
D60a	10.95	2	4	0	2	37	29	26
D57	11.3	4	6	0	2	25	23	40

Percentages of inertinite (including recycled vitrinite) and vitrinite macerals are typically below 10 vol.% (Table 2). Relatively high percentages of terrigenous macerals occur in marl samples below the CIE (e.g., D60; Fig. 4e). In contrast, terrigenous macerals occur in negligible amounts in most samples from the Toarcian CIE (Fig. 4). Primary vitrinite is rare in comparison with recycled vitrinite. Pyrite (mostly framboidal; e.g., Fig. 4f) is abundant in all samples, but rare in low-TOC samples below the CIE (D60, D62, D65) and in the uppermost sample (D128). Fish remains are abundant in some samples (e.g., D118). Vitrinite reflectance measurement gave values of 0.41 and 0.55%Rr for samples D127 and D115, respectively.

### Molecular composition of hydrocarbons

**Fig. 5:**
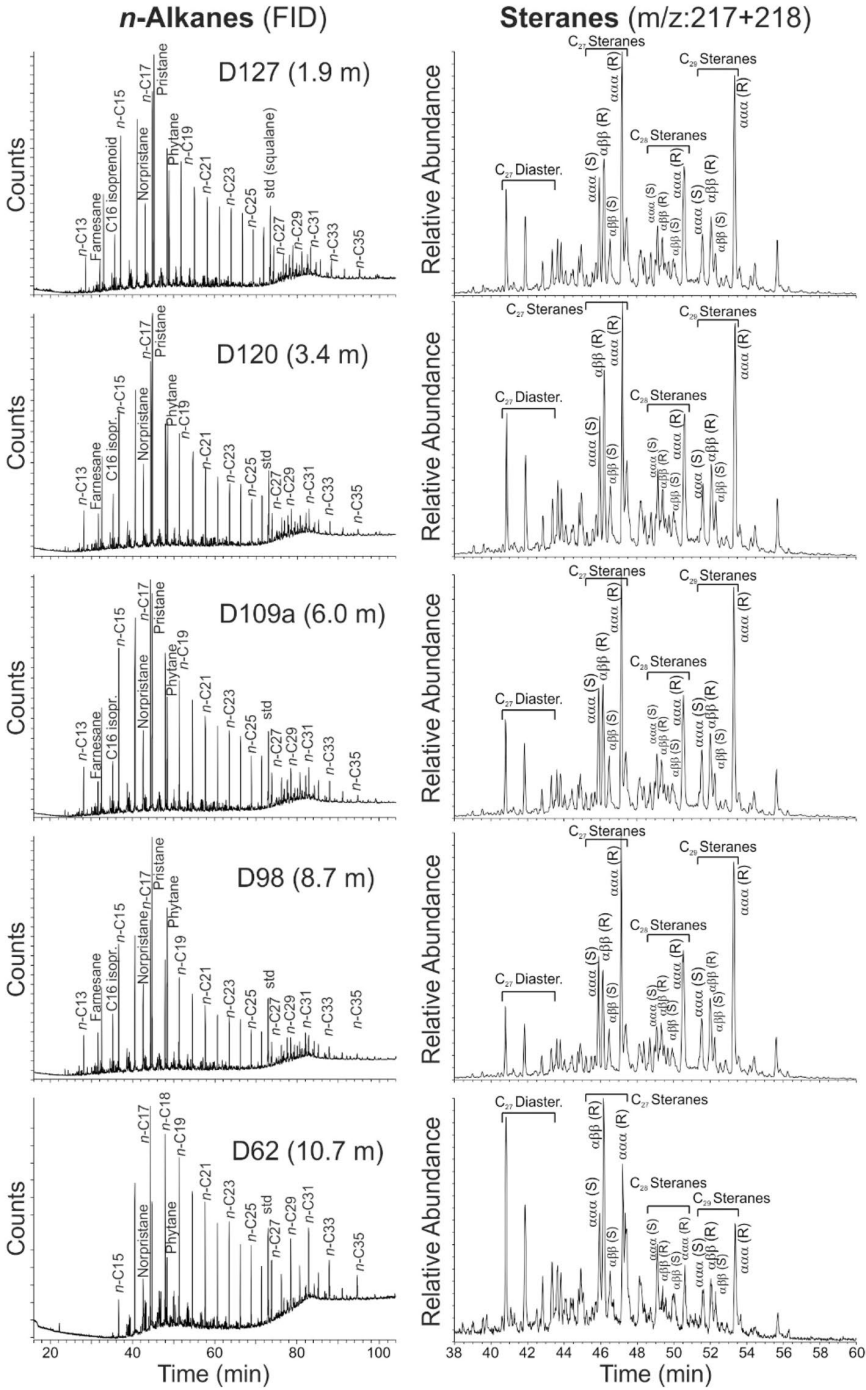
FID (flame ionization detector) traces of the saturated hydrocarbon fraction, and mass chromatogram representations of steranes of samples from different stratigraphic positions within the Dormettingen section

**Fig. 6:**
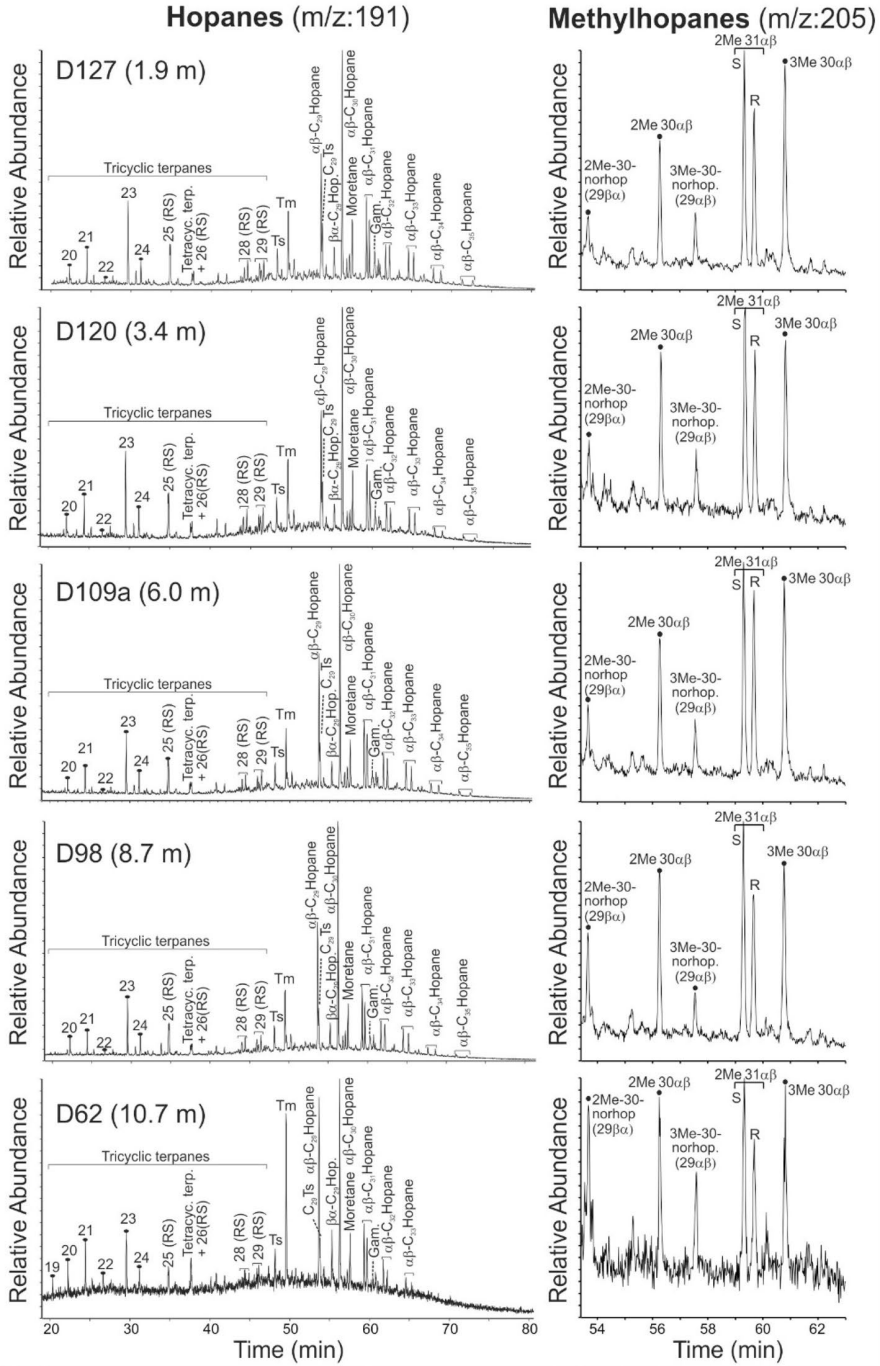
Mass chromatogram representations of hopanes and methylhopanes of samples from different stratigraphic positions within the Dormettingen section. 2Me-30-norhop—(29βα)-2α(CH_3_),17β(H),21a(H)-30-norhopane, 29αβ—2α(CH_3_),17α(H),21β(H)-hopane, 3Me-30-norhop (29αβ)—3β(CH_3_),17α(H),21β(H)-30-norhopane, 2Me 31αβ S—(22S)-2α(CH_3_),17α(H),21β(H)-29-homohopane, 2Me 31αβ R—(22R)-2α(CH_3_),17α(H),21β(H)-29-homohopane, 3Me 30αβ—3β(CH_3_),17α(H),21β(H)-30-hopane.

**Fig. 7:**
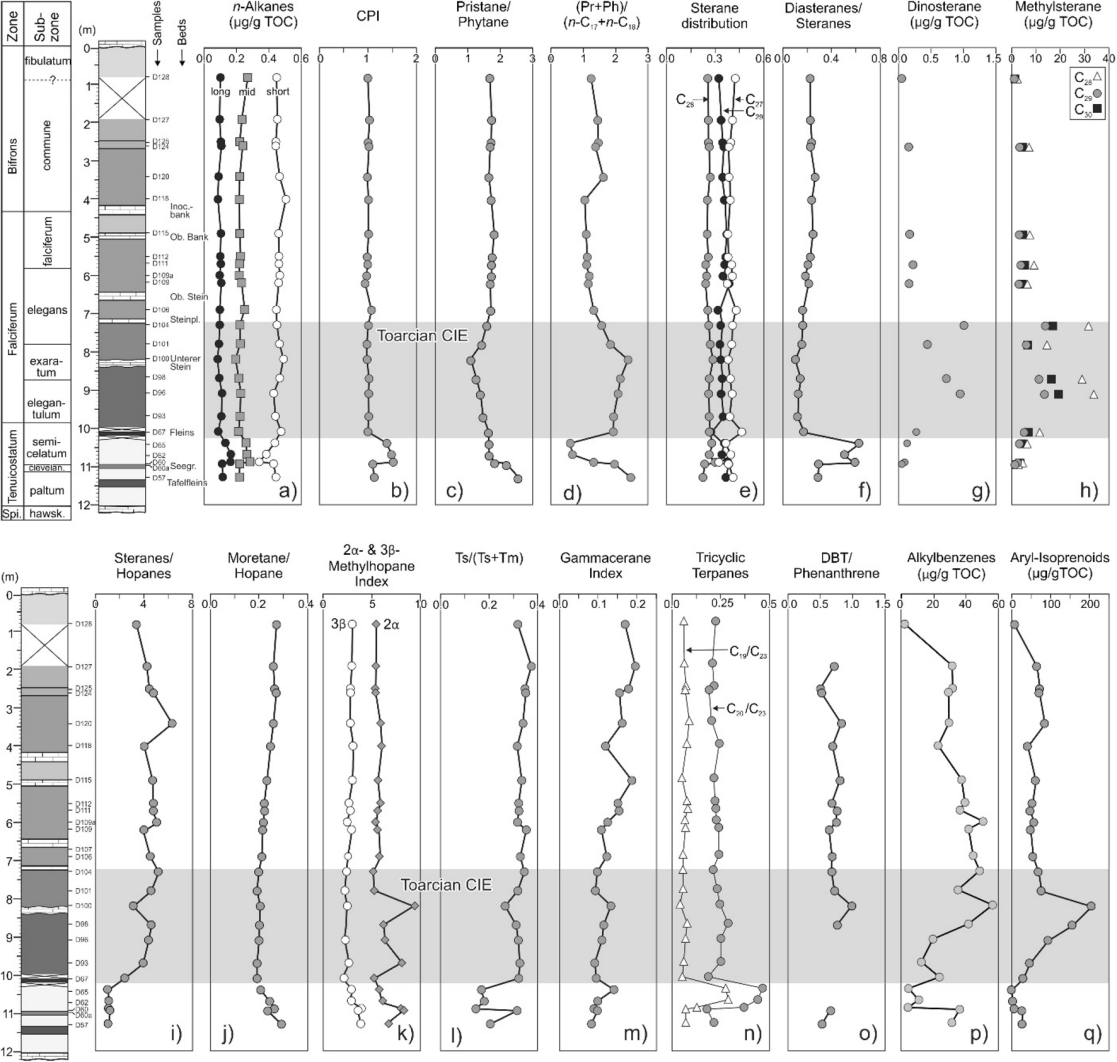
Concentrations and ratios of selected organic compounds at Dormettingen. The position of the negative carbon isotope excursion (CIE) is shown after Galasso et al. (2021). Alkane distribution: short-chain: *n*-C_15-19_/Σ*n*-alkanes; mid-chain: *n*-C_21-25_/Σ*n*-alkanes; long-chain: *n*-C_27-31_/Σ*n*-alkanes; CPI: Carbon Preference Index (Bray and Evans 1961), sterane distribution: C_27_/C_27-29_ steranes, C_28_/C_27-29_ steranes, C_29_/C_27-29_ steranes; DBT: dibenzothiophene

GC-FID and GC–MS traces of representative samples are presented in Figs. 5 and 6. Concentrations and ratios of selected organic compounds are listed in Table 3 and are plotted versus stratigraphic height in Fig. 7.

**Table 3:** Concentrations and concentration ratios of compounds and compound groups within the hydrocarbon fractions of Toarcian sediments from the Dormettingen section

Sample ID	Depth [m]	*n*-C_15–19_/	*n*-C_21–25_/	*n*-C_27–31_/	CPI	Pr/Ph	(Pr + Ph)/(*n*-C_17+18_)	Steranes [mg/g EOM]	C_27_/	C_28_/	C_29_/	S/(S + R)	αββ/(αββ+ααα)	C_27_ diasteranes/C_27_ sterane	Methylsteranes			Dino-steranes
		*n*-alkanes							C_27–29_ steranes			C_29_ steranes	C_29_ steranes		C_28_	C_29_	C_30_	
															[µg/g EOM]			
D128	0.8	0.45	0.27	0.10	1.00	1.77	1.25	46	0.42	0.25	0.32	0.21	0.29	0.23	2.23	1.01	1.27	0.05
D127	1.9	0.45	0.23	0.10	1.04	1.83	1.46	77	0.41	0.26	0.34	0.19	0.31	0.23	n.d	n.d	n.d	n.d
D125	2.49	0.44	0.22	0.10	1.01	1.81	1.48	75	0.40	0.26	0.35	0.20	0.31	0.24	n.d	n.d	n.d	n.d
D124	2.6	0.44	0.24	0.11	1.03	1.79	1.39	66	0.38	0.26	0.35	0.20	0.31	0.23	7.02	3.24	4.38	0.16
D120	3.4	0.46	0.22	0.09	0.99	1.74	1.63	80	0.38	0.27	0.35	0.23	0.32	0.27	n.d	n.d	n.d	n.d
D118	4	0.50	0.22	0.08	1.02	1.82	1.06	44	0.39	0.25	0.36	0.24	0.32	0.24	n.d	n.d	n.d	n.d
D115	4.9	0.46	0.22	0.10	1.02	1.91	1.10	72	0.38	0.25	0.37	0.23	0.33	0.26	7.37	3.03	4.55	0.18
D112	5.5	0.46	0.23	0.10	0.99	1.84	1.13	70	0.38	0.26	0.36	0.23	0.32	0.23	n.d	n.d	n.d	n.d
D111	5.7	0.46	0.22	0.10	1.00	1.82	1.11	77	0.40	0.24	0.36	0.23	0.32	0.21	9.10	3.65	5.30	0.23
D109a	6	0.47	0.22	0.10	0.98	1.82	1.20	97	0.41	0.25	0.35	0.23	0.33	0.19	n.d	n.d	n.d	n.d
D109	6.2	0.46	0.23	0.11	0.95	1.79	1.17	86	0.37	0.24	0.39	0.23	0.32	0.22	6.35	2.92	4.23	0.17
D106	6.9	0.44	0.25	0.10	1.08	1.80	1.33	91	0.43	0.25	0.32	0.23	0.31	0.17	n.d	n.d	n.d	n.d
D104	7.3	0.45	0.22	0.10	1.01	1.68	1.58	84	0.41	0.26	0.33	0.24	0.30	0.17	31.56	13.81	16.78	1.02
D101	7.8	0.46	0.22	0.09	0.98	1.52	1.85	50	0.40	0.27	0.33	0.22	0.30	0.16	14.42	5.96	6.50	0.45
D100	8.2	0.49	0.19	0.08	0.99	1.18	2.39	85	0.38	0.29	0.33	0.23	0.28	0.11	n.d	n.d	n.d	n.d
D98	8.7	0.47	0.21	0.09	1.03	1.35	2.15	92	0.39	0.26	0.34	0.22	0.31	0.15	28.90	11.18	16.28	0.75
D96	9.1	0.43	0.23	0.11	1.02	1.48	2.08	93	0.40	0.26	0.34	0.22	0.31	0.13	33.71	13.41	19.11	0.96
D93	9.7	0.44	0.22	0.11	1.02	1.56	1.95	91	0.39	0.26	0.35	0.22	0.31	0.13	n.d	n.d	n.d	n.d
D67	10.1	0.47	0.21	0.09	1.00	1.73	1.94	20	0.46	0.26	0.27	0.26	0.38	0.18	11.41	5.05	6.74	0.28
D65	10.4	0.44	0.26	0.13	1.39	1.75	0.61	3	0.37	0.28	0.36	0.22	0.36	0.63	6.30	3.07	4.28	0.14
D62	10.7	0.38	0.26	0.16	1.49	1.75	0.68	4	0.40	0.26	0.34	0.24	0.35	0.52	n.d	n.d	n.d	n.d
D60	10.9	0.34	0.28	0.16	1.52	1.92	1.33	11	0.32	0.30	0.38	0.28	0.37	0.60	4.42	2.33	3.35	0.10
D60a	10.95	0.43	0.22	0.11	1.10	2.28	1.97	36	0.38	0.23	0.38	0.27	0.36	0.30	2.68	1.28	1.81	0.06
D57	11.3	0.44	0.22	0.11	1.13	2.64	2.47	19	0.41	0.22	0.37	0.29	0.34	0.30	n.d	n.d	n.d	n.d

Sample ID	Depth [m]	Hopanes	Steranes/hopanes	S/(S + R)	Moretane/hopane	Homohopane index	2α	3β	Ts/(Ts + Tm)	Gammacerane/(Gamm. + C_30_ hopane)	Tricyclic terpanes		
				C_31_ hopanes			Methylhopane index				Σ	C_19_/C_23_	C_20_/C_23_
		[mg/g EOM]									[µg/g EOM]		
D128	0.8	14	3.37	0.59	0.27	0.04	5.67	3.12	0.32	0.17	5.44	0.03	0.20
D127	1.9	18	4.25	0.60	0.26	0.05	5.70	3.11	0.37	0.20	7.84	0.04	0.18
D125	2.49	17	4.43	0.60	0.26	0.04	5.54	2.90	0.35	0.18	7.17	0.04	0.19
D124	2.6	14	4.78	0.59	0.27	0.03	5.56	2.90	0.35	0.16	5.93	0.04	0.16
D120	3.4	13	6.32	0.59	0.26	0.04	6.10	2.90	0.34	0.16	6.62	0.06	0.18
D118	4	11	4.02	0.58	0.25	0.04	6.24	3.20	0.32	0.12	3.86	0.05	0.22
D115	4.9	15	4.71	0.59	0.23	0.04	5.89	3.15	0.33	0.19	5.29	0.02	0.19
D112	5.5	15	4.77	0.58	0.22	0.04	6.05	2.74	0.32	0.15	5.10	0.05	0.19
D111	5.7	16	4.77	0.59	0.22	0.04	5.84	2.93	0.32	0.15	5.67	0.05	0.20
D109a	6	19	5.04	0.57	0.22	0.03	5.54	2.56	0.32	0.12	7.55	0.04	0.20
D109	6.2	21	3.99	0.58	0.22	0.03	5.79	3.05	0.35	0.11	4.98	0.04	0.21
D106	6.9	20	4.51	0.58	0.21	0.03	5.94	2.63	0.33	0.12	6.98	0.03	0.22
D104	7.3	16	5.17	0.59	0.20	0.04	5.27	2.48	0.34	0.10	7.28	0.03	0.18
D101	7.8	11	4.57	0.58	0.19	0.03	5.37	2.29	0.32	0.09	4.68	0.03	0.21
D100	8.2	27	3.10	0.60	0.21	0.03	9.67	2.49	0.27	0.13	14.02	0.01	0.22
D98	8.7	20	4.59	0.58	0.20	0.04	6.37	2.43	0.31	0.11	7.43	0.05	0.26
D96	9.1	21	4.37	0.59	0.20	0.03	6.53	2.30	0.32	0.11	8.02	0.04	0.22
D93	9.7	23	3.93	0.59	0.19	0.03	8.31	2.67	0.33	0.09	7.86	0.03	0.22
D67	10.1	8	2.40	0.62	0.19	0.04	5.35	2.16	0.32	0.10	5.74	0.03	0.16
D65	10.4	3	0.98	0.69	0.21	0.02	5.77	2.91	0.17	0.14	0.77	0.25	0.44
D62	10.7	4	1.07	0.59	0.24	0.01	6.11	2.91	0.18	0.10	0.84	0.26	0.42
D60	10.9	11	1.05	0.61	0.26	0.00	8.04	3.98	0.15	0.09	2.68	0.10	0.34
D60a	10.95	30	1.18	0.59	0.24	0.05	8.70	3.78	0.32	0.10	6.35	0.04	0.15
D57	11.3	19	1.01	0.59	0.29	0.04	6.88	3.96	0.20	0.08	4.15	0.04	0.19

Sample ID	Depth [m]	MP	DMP	MPI-1	Rc [%]	MDBT [µg/g EOM]	MDR	DBT/Phen	Methylated alkylbenzenes [µg/g EOM]	Aryl-isoprenoids [µg/g EOM]	AIR C_13–17_/C_18-22_
		[µg/g EOM]									
D128	0.8	n.d	n.d	n.d	n.d	n.d	0.32	n.d	2.07	9.51	0.08
D127	1.9	9.59	20.18	0.36	0.62	10.55	0.32	0.74	31.51	68.14	0.45
D125	2.49	12.43	19.87	0.46	0.67	11.88	0.31	0.53	31.75	75.81	0.55
D124	2.6	9.13	20.56	0.40	0.64	11.66	0.26	0.55	29.22	74.48	0.33
D120	3.4	9.44	12.96	0.17	0.50	12.92	0.29	0.85	29.53	88.77	0.45
D118	4	10.97	18.93	0.39	0.64	12.12	0.35	0.72	22.76	44.07	0.48
D115	4.9	12.19	23.47	0.28	0.57	11.38	0.36	0.83	37.31	64.88	0.49
D112	5.5	13.57	18.91	0.31	0.58	13.20	0.35	0.71	39.40	56.13	0.55
D111	5.7	9.85	9.25	0.19	0.51	12.52	0.38	0.79	36.30	50.87	0.60
D109a	6	12.33	20.45	0.32	0.59	15.00	0.36	0.78	50.65	60.56	0.80
D109	6.2	12.68	24.69	0.33	0.60	14.26	0.33	0.66	41.69	51.74	0.46
D106	6.9	13.99	12.37	0.25	0.55	13.81	0.35	0.71	44.49	58.09	0.76
D104	7.3	12.10	15.22	0.29	0.57	12.16	0.32	0.70	48.48	72.28	0.70
D101	7.8	10.22	15.66	0.43	0.66	10.53	0.30	0.75	35.08	80.06	0.59
D100	8.2	14.75	12.47	0.51	0.70	14.45	0.29	1.02	56.52	210.86	0.41
D98	8.7	18.94	8.20	0.52	0.71	14.20	0.29	0.79	41.57	160.77	0.41
D96	9.1	0.00	12.49	n.d	n.d	n.d	n.d	n.d	19.56	97.47	0.49
D93	9.7	0.00	7.50	n.d	n.d	n.d	n.d	n.d	12.42	49.53	0.43
D67	10.1	0.00	11.67	n.d	n.d	n.d	n.d	n.d	23.57	32.26	0.41
D65	10.4	n.d	n.d	n.d	n.d	n.d	n.d	n.d	4.39	1.79	n.d
D62	10.7	n.d	n.d	n.d	n.d	n.d	n.d	n.d	10.83	4.98	n.d
D60	10.9	n.d	n.d	n.d	n.d	n.d	n.d	0.22	4.09	6.92	n.d
D60a	10.95	12.30	19.67	0.24	0.54	13.10	0.33	0.68	36.15	29.24	1.25
D57	11.3	16.50	25.94	0.68	0.81	14.74	0.34	0.56	31.29	29.98	1.02

*EOM* extractable organic matter, *CPI* carbon preference index (Bray and Evans 1961), *Pr* pristine, *Ph* phytane, *M* methylphenanthrene, *DMP* dimethylphenanthrene, *MPI* methylphenanthrene index (Radke et al. 1986), *MDBT* methyldibenzothiophene, *MDR* methyldibenzothiophene ratio (Radke et al. 1986), *Rc* calculated vitrinite reflectance, *DBT* dibenzothiophene, *Phen* phenanthrene, *AIR* aryl isoprenoid ratio (Schwark and Frimmel 2004)

#### n-Alkanes and isoprenoids

*n*-Alkanes are abundant in all samples and show broad chain length distributions (*n-*C_13–35_) (Fig. 5). Short-chain *n*-alkanes are always dominating (*n-*C_15–19_/∑*n*-alkanes: 0.34–0.50) over mid- (*n-*C_21–25_/∑n-alkanes: 0.19–0.28) and long-chain *n*-alkanes (*n-*C_27–31_/∑*n*-alkanes: 0.08–0.16) (Fig. 7a). Three low-TOC samples below the Toarcian CIE (D60, D62, D65) show relative high concentrations of mid- and long-chain *n*-alkanes. The carbon preference index (CPI; according to Bray and Evans 1961) is close to 1 (0.95–1.13) in all samples except for the three low-TOC samples mentioned above (1.39–1.52) (Figs. 5, 7b).

The pristane/phytane (Pr/Ph) ratio (e.g., Didyk et al. 1978) decreases upwards in the lower part of the section (11.3–8.2 m) from 2.6 to 1.8 and reaches a minimum value in the upper part of the *exaratum* Subzone (D100). Between samples D100 (*exaratum* Subzone) and D106 *elegans* Subzone), Pr/Ph ratios increase again and are fairly constant (1.7–1.9) in the upper part of the section (Fig. 7c).

Below the Toarcian CIE, the ratio of pristane and phytane versus *n*-C_17_ and *n*-C_18_ (e.g., Frimmel et al. 2004) decreases upwards and shows an opposite trend to the Pr/Ph ratio in the Toarcian CIE and the remaining part of the *falciferum* Zone (Fig. 7d). An increase is observed above the Inoceramenbank between samples D118 and D120. The uppermost part of the section is characterized by a constant decrease.

#### Steroids

Sterane concentrations range from 4 to 96 mg/g EOM (extractable organic matter). C_27_ steranes dominate in all samples closely followed by C_29_ steranes (Fig. 5; Table 3). C_28_ steranes are typically less abundant, but relatively high concentrations are observed in samples D109a to D115 within the *falciferum* Subzone (Fig. 7e). The C_29_ sterane 20S/(20S + 20R) ratio and C_29_ sterane αββ/(αββ + ααα) ratio are maturity parameters (Seifert and Moldowan 1986) and range from 0.19 to 0.29 (avg. 0.23) and from 0.28 to 0.38 (avg. 0.32), respectively. Both ratios show a subtle upward decrease (Table 3).

C_27_ diasteranes are present in all samples in lower amounts than their C_27_ regular sterane counterparts (Fig. 5). The C_27_ diasterane/C_27_ regular sterane ratio varies between 0.11 and 0.63 and is especially high in samples below the CIE (Fig. 7f).

**Fig. 8:**
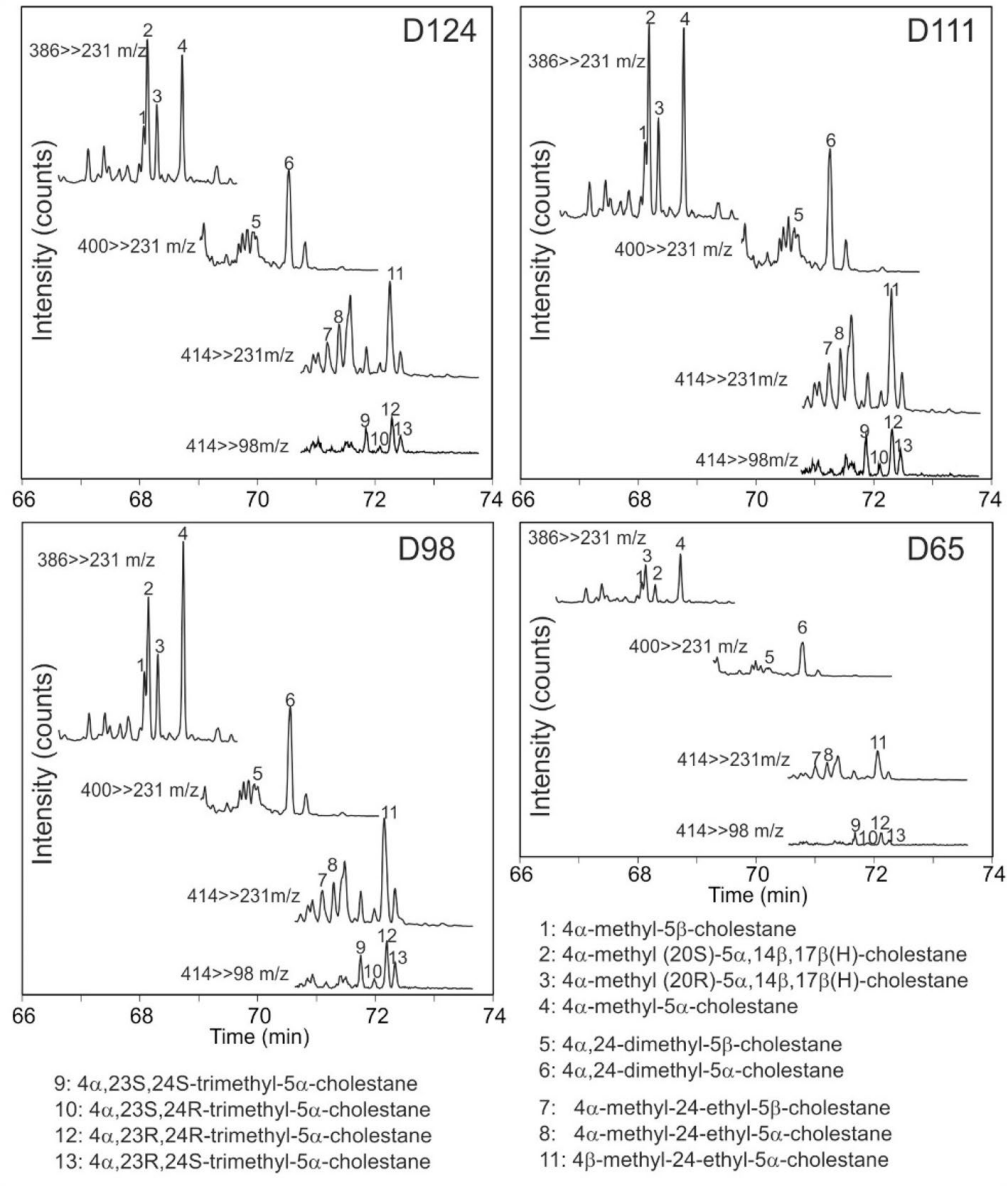
4-methylsterane distributions of four samples. Data were acquired by GC-MSMS. Identification of 4α-methylsteranes, 4β-methylsteranes is based on Goodwin et al. (1988) and Schouten et al. (2000)

4-Methylsteranes (C_28_: 4α-methyl-5α-cholestane; C_29_: 4α,24-dimethyl-5α-cholestane; C_30_: 4α-methyl-24-ethyl-5α-cholestane) and dinosterane (4α,23S,24R-trimethyl-5α-cholestane) have been detected in many samples (Fig. 8). The highest concentrations occur in the CIE interval (Table 3; Fig. 7g, h).

#### Hopanoids and related compounds

The dominant non-aromatic cyclic triterpenoids are hopanes (3–30 mg/g EOM) (Fig. 6; Table 3). The sterane/hopane ratio shows an upward increase with a maximum in sample D120 followed by a decrease (Fig. 7i). Concentrations of C_31_ to C_35_ 17α,21β(H)-homohopanes decrease with increasing carbon number (Fig. 6). 22S/(22S + 22R) isomer ratios of αβ C_31_ hopane range from 0.57 to 0.61 (Table 3). The moretane/hopane ratio ranges from 0.19 to 0.29 and is relatively low (≤ 0.21) within the CIE (Fig. 7j).

The C_35_ homohopane index (HHI = C_35_/(C_31_–C_35_) homohopanes; Peters and Moldowan 2001) is below 0.02 for low-TOC samples below the CIE and 0.3 to 0.5 for the rest of the samples without a specific depth trend (Table 3).

**Fig. 9:**
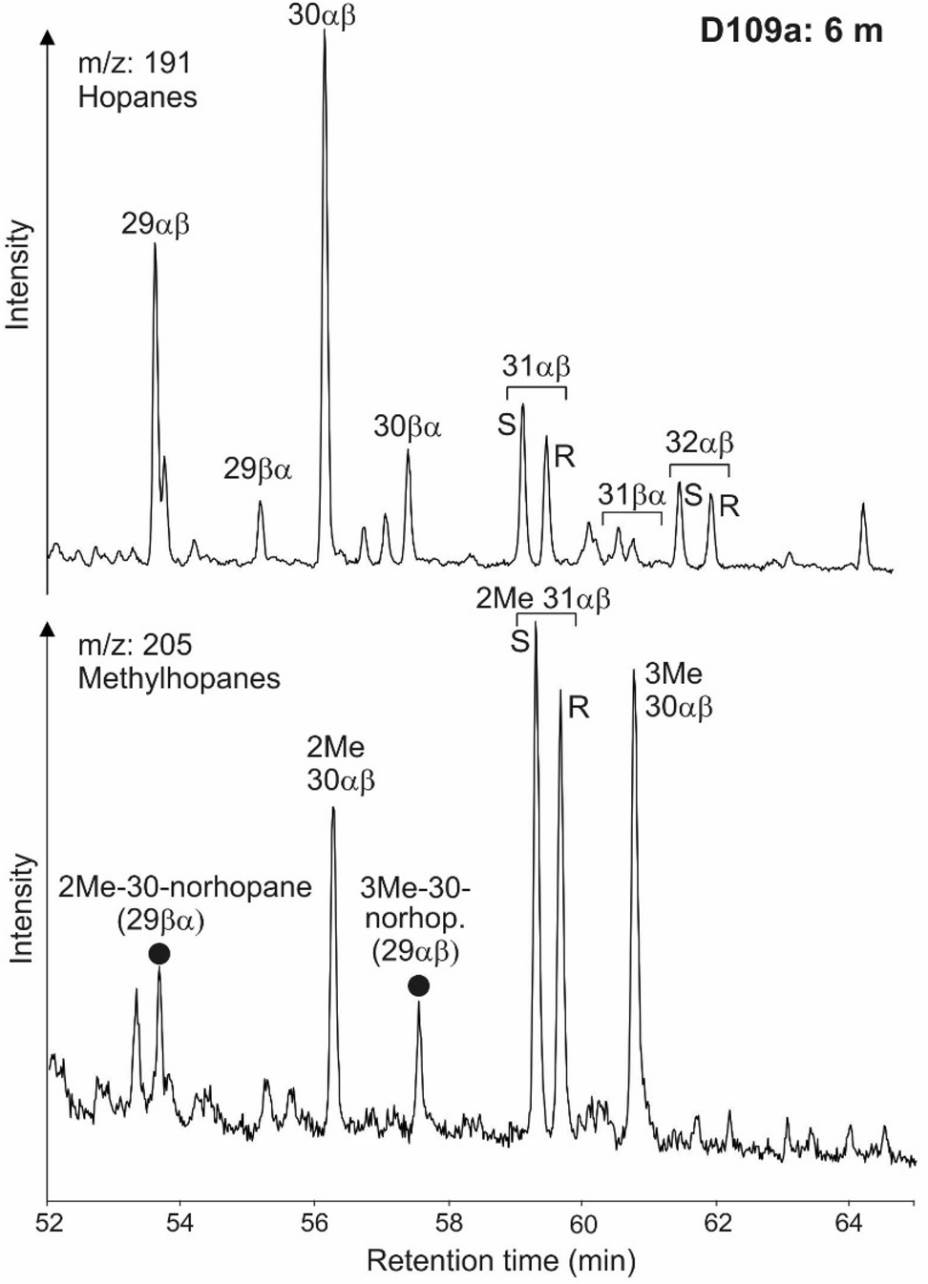
Mass chromatograms of *m/z* 191 and 205 showing hopane and methylhopane distribution of sample D109a. Methylhopane peak identification of 2Me and 3Me is based on Farrimond et al. (2004) and Pancost et al. (1998). 2Me-30-norhop—(29βα)-2α(CH_3_),17β(H),21a(H)-30-norhopane, 29αβ—2α(CH_3_),17α(H),21β(H)-hopane, 3Me-30-norhop (29αβ)—3β(CH_3_),17α(H),21β(H)-30-norhopane, 2Me 31αβ S—(22S)-2α(CH_3_),17α(H),21β(H)-29-homohopane, 2Me 31αβ R—(22R)-2α(CH_3_),17α(H),21β(H)-29-homohopane, 3Me 30αβ—3β(CH_3_),17α(H),21β(H)-30-hopane

Methylhopanes occur in significant amounts in the studied samples and have been identified following Farrimond et al. (2004) and Pancost et al. (1998) (Figs. 7, 9). A minor overlap with regular hopanes occurs for some methylhopanes. Hence, a slight overestimation of the methylhopane concentrations cannot be excluded (Fig. 9, see also Farrimond et al. 2004). The 2α-methylhopane index (2α-MHI; 2α-methylhopane/[2α-methylhopane + hopanes]) and the 3β-methylhopane index (3β-MHI; 3β-methylhopane/[3β-methylhopane + hopanes]) have been calculated according to Summons et al. (1999) and Jiao et al. (2008). The 2α-MHI shows two maxima in the Toarcian CIE, while the 3β-MHI is slightly elevated below the CIE, but overall relatively constant (Fig. 7k).

The Ts/(Ts + Tm) ratio varies between 0.15 and 0.37 (Fig. 7l). The gammacerane index (gammacerane/(gammacerane + C_30_ hopane) (Sinninghe Damsté et al. 1995) ranges from 0.1 to 0.2 and increases upwards (Fig. 7m).

Apart from hopanes, tricyclic terpanes (TT) also occur with significant concentrations (0.77–14.02 μg/g EOM; Fig. 6). C_19_ to C_29_ TTs are present, but C_27_ TT is absent_._ The dominant TT is C_23_ TT as reflected by low C_19_/C_23_ TT (0.01–0.26) and C_20_/C_23_ TT ratios (0.15–0.44) (Table 3). Depth plot shows that low-TOC samples below the CIE (D60, D62, D65) are characterized by relatively high C_19_/C_23_ TT and C_20_/C_23_ TT ratios (Fig. 7n).

#### Aromatic hydrocarbons

The Posidonia Shale contains benzenes, napthalenes, phenanthrenes, dibenzothiophenes, and their alkylated equivalents in the aromatic fraction. Alkylphenanthrenes are present and include methylphenanthrene (MP), and dimethylphenanthrene (DMP). The methylphenanthrene index (MPI-1; Radke et al. 1986; Radke 1988) ranges from 0.17 to 0.68, but could not be determined for all samples.

**Fig. 10:**
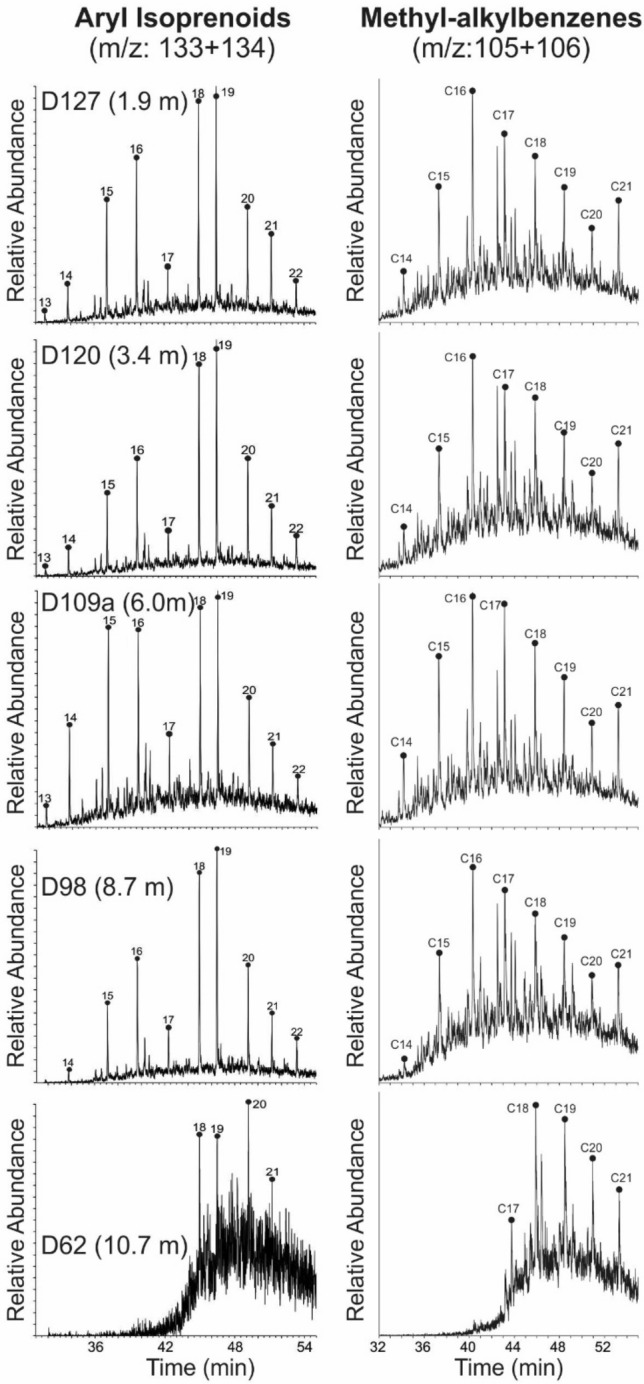
Mass chromatogram representation of aryl isoprenoids and methyl-*n*-alkylbenzenes. Carbon number over the peaks refers total carbon in alkylbenzenes

Concentrations of methyldibenzothiophene range from 10.5 to 15.0 μg/g EOM (avg. 12.9). The methyldibenzothiophene ratio (MDR; Radke et al. 1986) varies between 0.26 and 0.38. Dibenzothiophene (DBT) and phenanthrene (Phen) contents are also high with DBT/Phen ratios (Hughes et al. 1995; Fig. 7p) ranging from 0.22 to 1.01 (avg. 0.70; Table 3). Methyl-*n*-alkylbenzenes (e.g., Zhang et al., 2014) are present in considerable amounts in the aromatic fraction (2.1–56.5 μg/g EOM; avg. 31.2; Table 3) and reach maximum concentrations within the CIE (8.2 m; D100; Fig. 10).

**Fig. 11:**
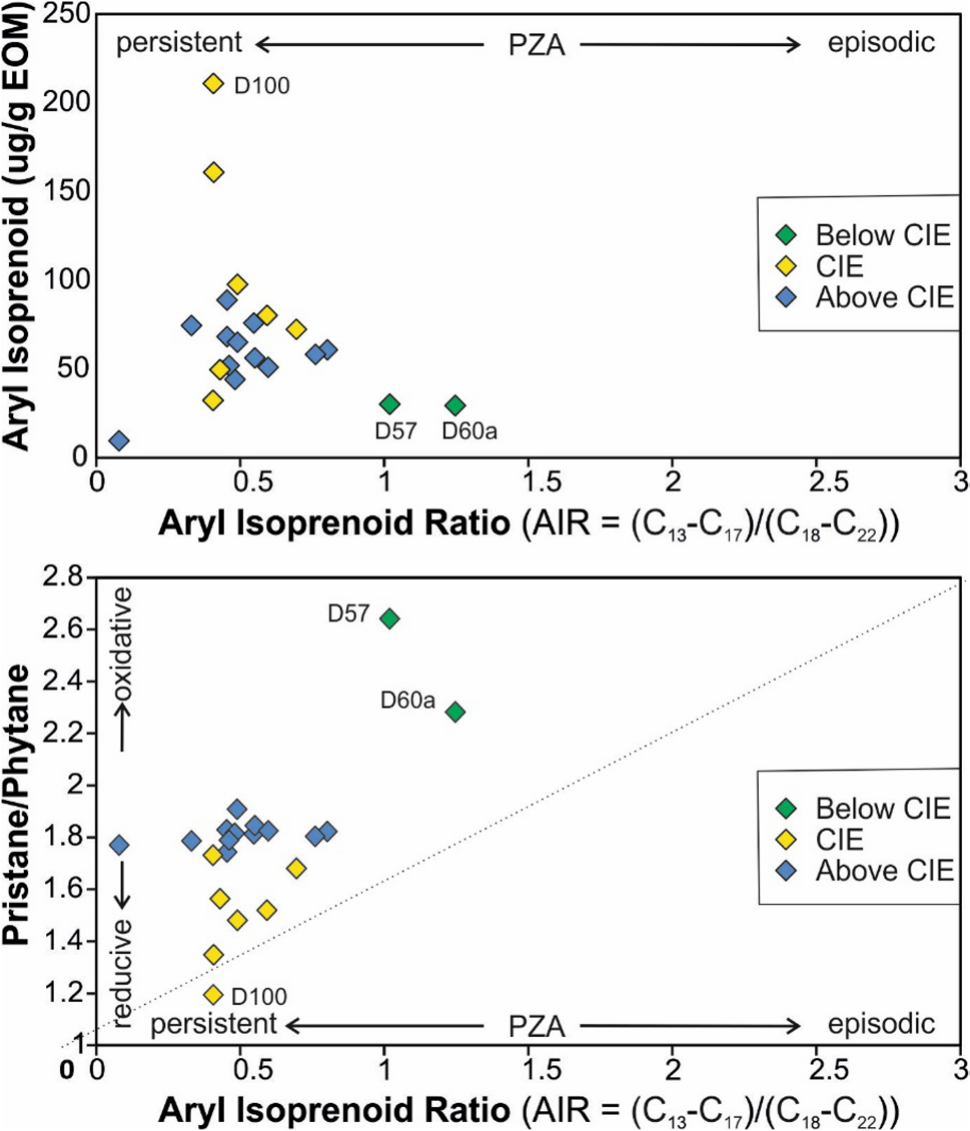
Redox conditions (photic zone anoxia) represented by aryl isoprenoid versus aryl isoprenoid ratio (C_13–17_/C_18–22_) and pristane/phytane ratio versus aryl isoprenoid ratio (C_13–17_/C_18–22_) (after Schwark and Frimmel 2004)

C_13_ to C_22_ aryl isoprenoids are observed in significant amounts in most samples. The maximum peaks are formed by C_18_ and C_19_ aryl isoprenoid (Fig. 10). Concentrations of aryl isoprenoids increase upwards in the lower part of the CIE and reach a maximum (210 µg/g EOM) in sample D100 (Fig. 7q). Above sample D100, aryl isoprenoid concentrations are lower and decrease gradually towards the top of the section. The ratio between C_13-17_ and C_18-22_ aryl isoprenoids (aryl isoprenoid ratios (AIR) following Schwark and Frimmel 2004) are typically low and exceed 1 only in the Tafelfleins (D57) and the Seegrasschiefer (D60a) below the CIE (Fig. 11). However, AIR could not be determined reliably for samples D60 to D65. In contrast to aryl isoprenoids, isorenieratane was not detected.

### Stable carbon isotope composition of organic matter

**Fig. 12:**
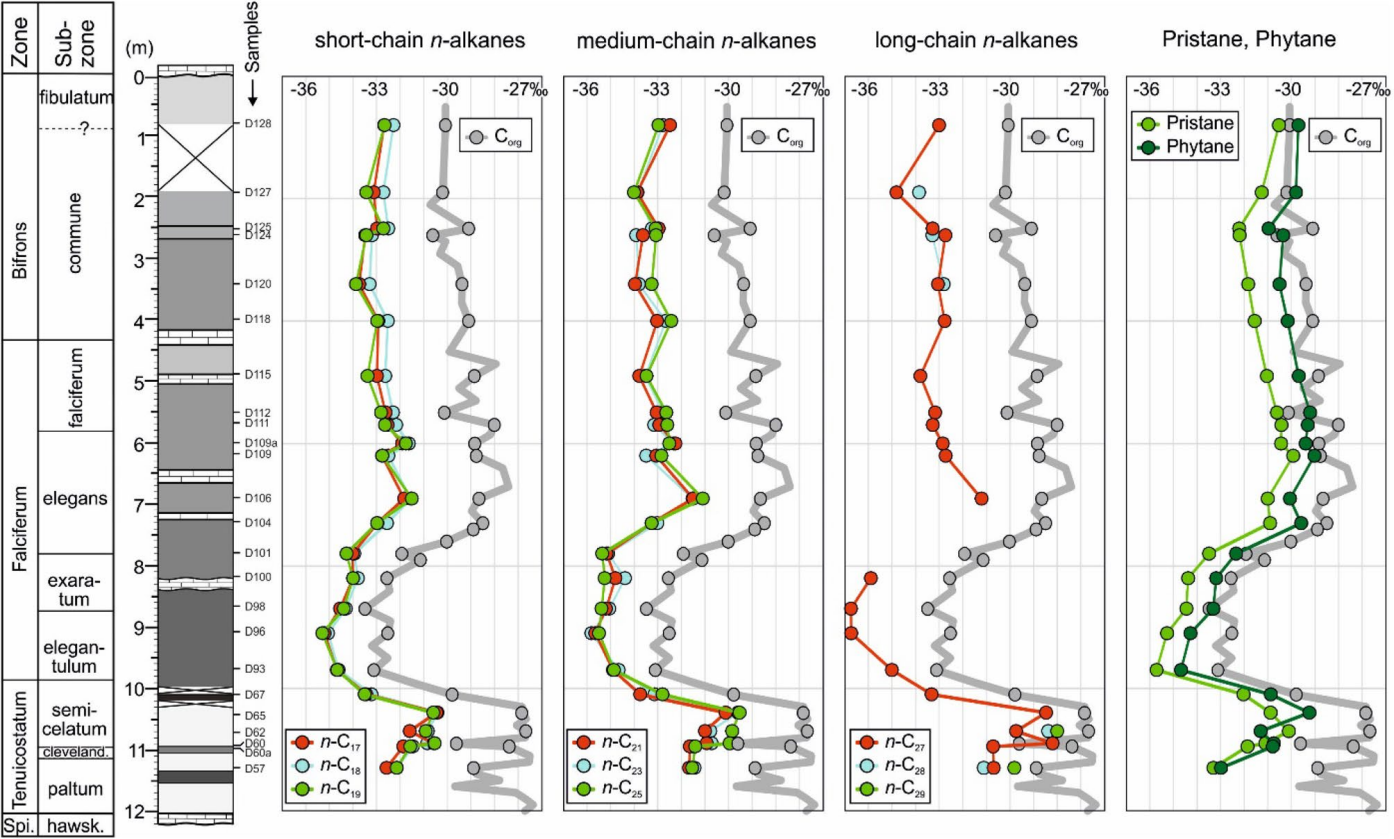
Depth plot representation of variation in δ^13^C of *n*-alkanes, pristane and phytane. Vertical variation of bulk carbon isotope values (ẟ^13^C_org_) is shown for comparison after Galasso et al. (2021)

Compound-specific carbon isotope ratios have been determined for *n*-alkanes with chain length ranging from C_15_ to C_29_ as well as for norpristane, pristane and phytane. Stratigraphic variations of δ^13^C values of individual compounds are presented in Fig. 12. It is obvious that the negative CIE is visible in all compounds. The strongest negative isotope shift is observed for *n*-C_27_. Reliable δ^13^C values for *n*-C_28_ and *n*-C_29_ could be obtained only for a few samples. However, these data show a similar strong shift. In contrast, the shift is less prominent for *n*-alkanes in the range of *n*-C_17_ to *n*-C_25_. Pristane and phytane display similar trends. With the exception of sample D62, carbon in phytane is isotopically slightly heavier than that in pristane. The most negative δ^13^C value for pristane and phytane is recorded for sample D93, near the base of the CIE (9.7 m), whereas the most negative δ^13^C values for *n*-alkanes (and C_org_; Galasso et al. 2021) are found in its middle part (samples D96 and D98).

**Fig. 13:**
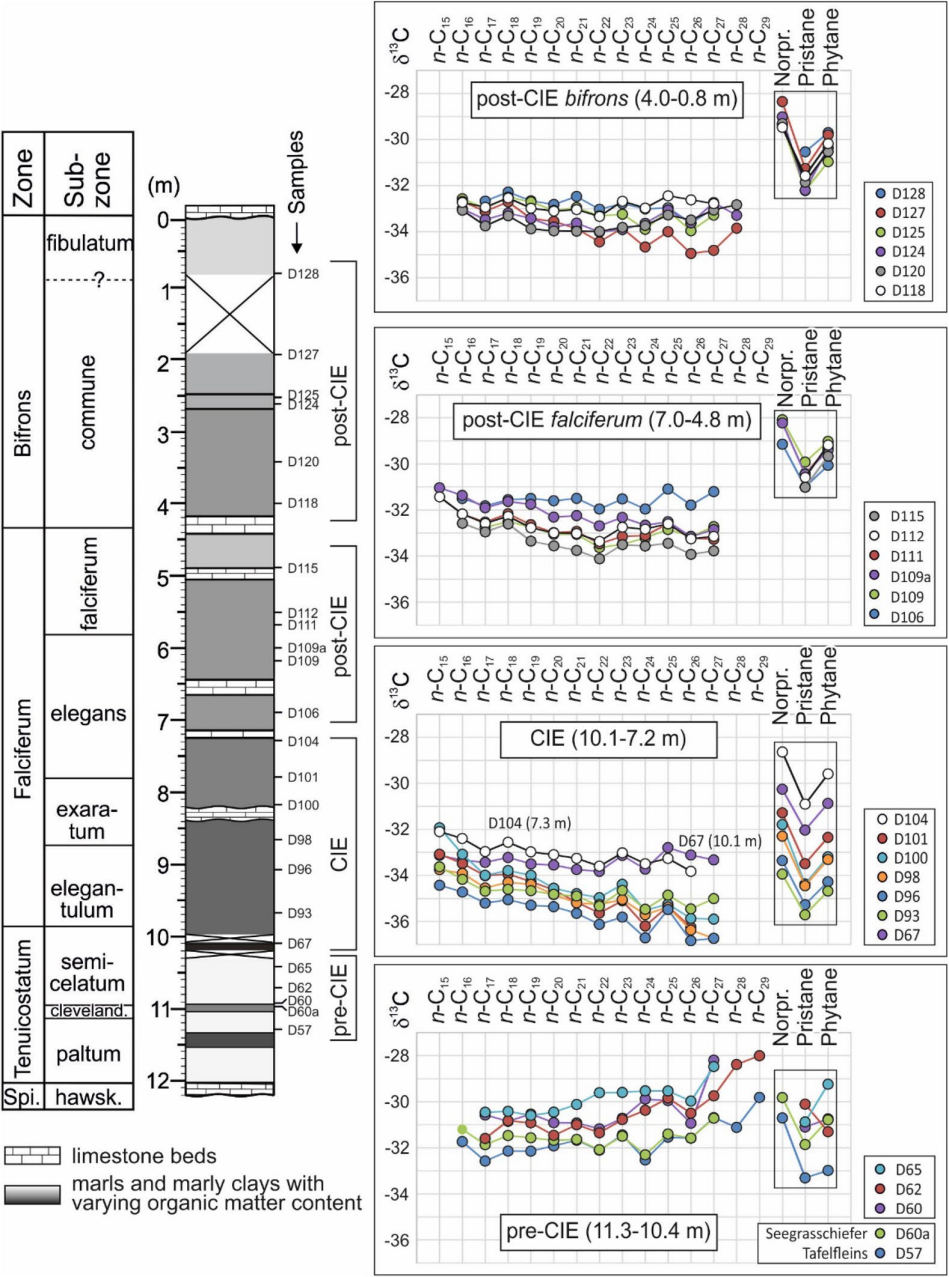
Carbon isotope composition of individual *n*-alkanes and isoprenoids for samples from different stratigraphic intervals in the Dormettingen section

Figure 13 shows plots of δ^13^C values versus chain length of *n*-alkanes. Apart from the differences in absolute values (lower values compared to δ^13^C_org_), samples from different depth intervals show different isotope patterns:

- Samples below the CIE (11.3–10.4 m) are characterized by a trend towards less negative δ^13^C values with increasing chain length. This trend is observed both, in organic-lean sediments and in organic-rich Tafelfleins and Seegrasschiefer samples. δ^13^C values of isoprenoids are similar to those of long-chain *n*-alkanes or even more negative.
- Samples within the CIE (10.1–7.8 m) show very negative δ^13^C values, which decrease with increasing chain length. Only the lowermost (D67) and uppermost sample (D104) show more or less constant δ^13^C values. δ^13^C values of isoprenoids are typically heavier than n-alkanes in this interval.
- Samples above the CIE show either a negative trend (10.1–7.8 m; 2.6–0.8 m) or rather constant values (5.5–3.4 m) whereby n-C_22_ often shows the most negative δ^13^C value. Isotope ratios of isoprenoids in these intervals are rather uniform and less negative than *n*-alkanes.

## Discussion

### Thermal maturity

Vitrinite reflectance (0.40–0.54%Rr), *T*_max_ (avg. 427 °C), and the low isomerization ratio of C_29_ steranes (avg. 0.23) suggest that the Dormettingen section is thermally immature. In contrast, early oil window maturity is suggested by the αββ/(αββ + ααα) ratios of C_29_ steranes (avg. 0.32) and the C_31_ hopane isomerisation values, which are at equilibrium (avg. 0.6).

**Fig. 14:**
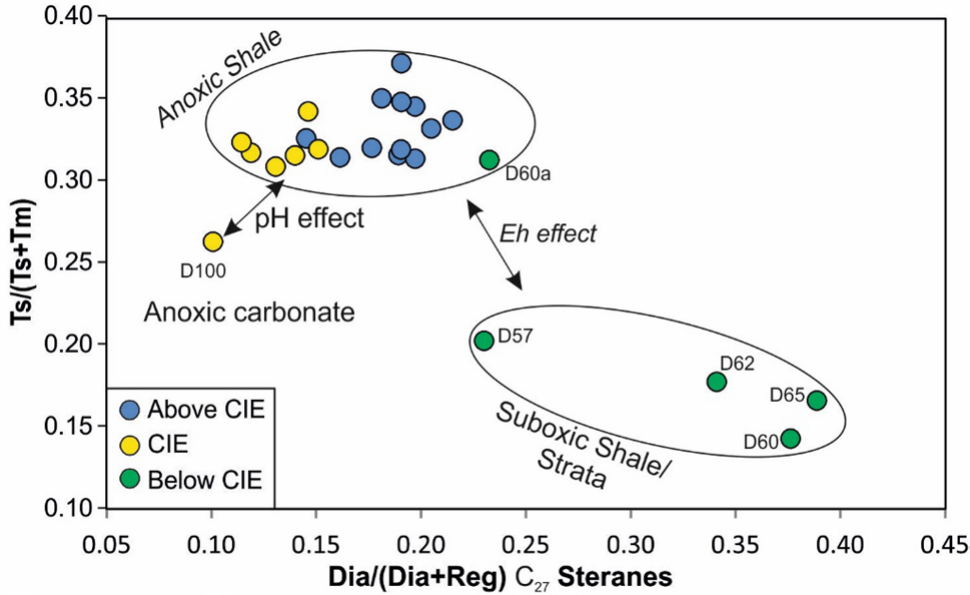
C_27_ diasteranes/C_27_ regular sterane versus Ts/(Ts + Tm) (plot after Moldowan et al. 1994). In case of the immature Posidonia Shale, differences in Ts/(Ts + Tm) ratios are not controlled by maturity but the depositional environment (Moldowan et al. 1994)

Maturity variations cannot be expected within the thin (~ 12 m) stratigraphic interval. All depth trends of maturity parameters [e.g., upward increase in *T*_max_; slight upward decrease of sterane isomerisation ratios; low moretane/hopane ratios in the CIE (Fig. 7j); low Ts/(Ts + Tm) below the CIE (Figs. 7l, 14)] reflect facies variations (see also Moldowan et al. 1986, 1994). Similar thermal maturity values were determined for the neighboring Dotternhausen section (Schmid-Röhl 1999; Frimmel et al. 2004).

### Depositional environment

In the following, geochemical proxies are used to reconstruct the depositional environment of the Dormettingen section considering the wealth of information provided by previous studies (van Acken et al. 2019; Galasso et al. 2021), including those on the Dotternhausen section (e.g., Röhl et al. 2001; Schmid-Röhl et al. 2002; Frimmel et al. 2004; Schwark and Frimmel 2004; Röhl and Schmid-Röhl 2005; van den Schootbrugge et al. 2005; Bour et al. 2007; Wang et al. 2020, 2021).

**Fig. 15:**
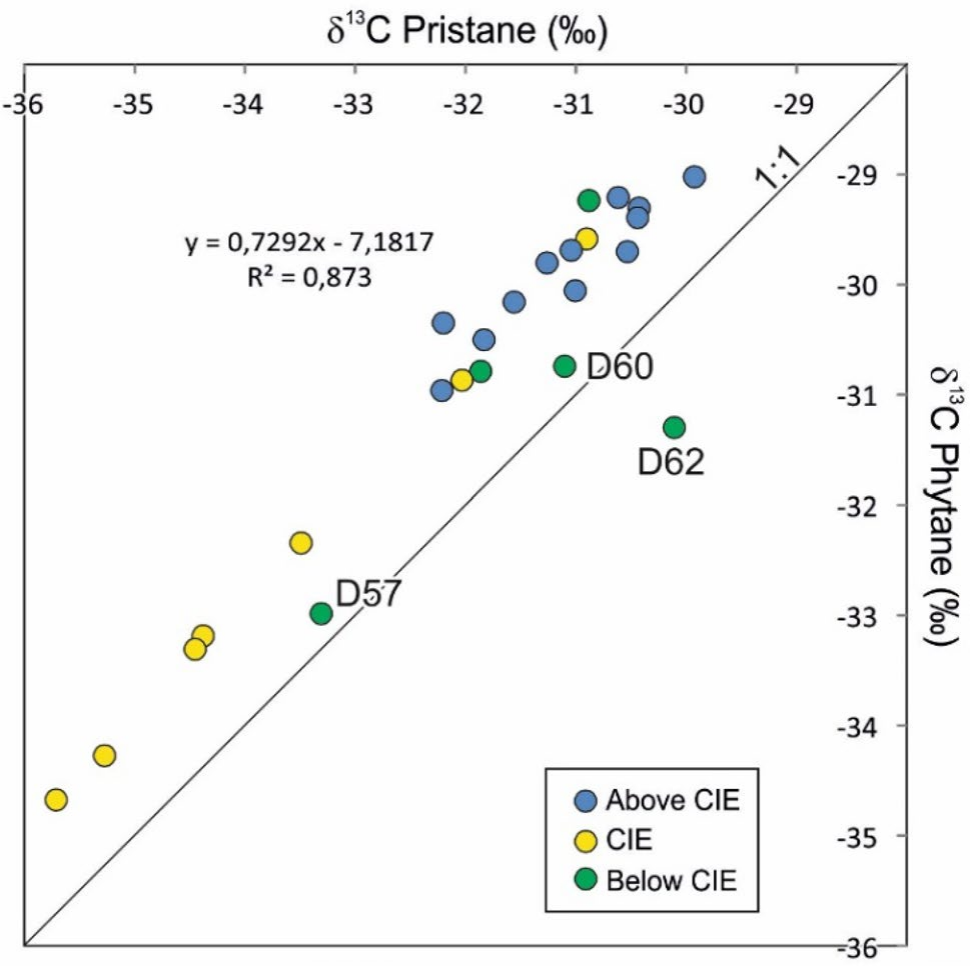
Cross-plot of δ^13^C values of pristane and phytane. δ^13^C values of pristane are generally about 1‰ more negative than δ^13^C values of phytane. No good correlation exists for samples below the Toarcian CIE

Before discussing the depositional environment of individual units, geochemical parameters based on pristane and phytane will be examined. The Pr/Ph ratio, a widely used redox proxy (e.g., Didyk et al. 1978), is unusually high in the Posidonia Shale of the SWGB (e.g., Schouten et al. 2000; Frimmel et al. 2004). Nevertheless, Frimmel et al. (2004) found a clear relationship between Pr/Ph and (Pr + Ph)/(*n*-C_17_ + *n*-C_18_) ratios with various paleoecological redox indicators. They showed that strictly anoxic conditions are associated with Pr/Ph ratios < 1.6 and (Pr + Ph)/(*n*-C_17_ + *n*-C_18_) ratios > 1.75. Environments with very short (weeks, month) and short (month, years) oxygen supply to the sediment surface are related to Pr/Ph ratios of about 1.7 and 1.75, respectively. The corresponding values for the (Pr + Ph)/(*n*-C_17_ + *n*-C_18_) ratios are ~ 1.6 and ~ 1.3. The unusual high Pr/Ph ratios were attributed to an additional input of pristane from tocopherols or chromans (Frimmel et al. 2004). Since tocopherols are formed by the same biochemical pathway as chlorophyll, tocopherol-derived pristane has the same δ^13^C as the phytol side chain (Hughes et al. 1995; Peters et al. 2005). This also applies to the chromans (Grice et al. 1998; Zhang et al. 2012). Thus, it is not surprising that (with the exception of samples below the Toarcian CIE) δ^13^C_pristane_ and δ^13^C_phytane_ show an excellent correlation (Fig. 15).

#### Pre-CIE (12.0–10.2m)

The *tenuicostatum* Zone is dominated by light-grey marls (Blaugraue Mergel; Aschgraue Mergel) with low-TOC contents (< 0.9 wt.%; Fig. 3). An oxygenated environment is indicated by bioturbation and the presence of a relatively diverse benthic macrofauna (Schmid-Röhl et al. 2002; Frimmel et al. 2004; Schwark and Frimmel 2004; Röhl and Schmid-Röhl 2005; van Acken et al. 2019). HI values (53–162 mgHC/gTOC) reflect the poor preservation conditions and/or suggest a high contribution of terrigenous organic matter. Indeed, a significant contribution of terrigenous phytoclasts (up to 65%) is characteristic for this interval (palynofacies A of Galasso et al. 2021). A high amount of terrigenous organic matter is also indicated by maceral analysis and geochemical proxies. The latter include elevated amounts of long-chain *n*-alkanes, high CPI values (Bray and Evans 1961), and high ratios of C_19_/C_23_ and C_20_/C_23_ tricyclic terpanes (Noble et al. 1986; Peters et al. 2005; French et al. 2014; Fig. 7a, b, n). HI values of the light-grey marls increase upwards (Fig. 3). Since the ratio of aquatic to terrigenous phytoclasts does not vary in a systematic way (Galasso et al. 2021), the observed HI values probably reflect a trend towards better preservation conditions. In addition, the upward decreasing Pr/Ph ratios (Fig. 7c) support a general trend towards less oxic conditions. Oxic to suboxic conditions are also reflected by very low values of the C_35_ homohopane index (C_35_ HHI ≤ 0.02) (Table 3).

High TOC contents (3.6–11.1 wt.%) in the *tenuicostatum* Zone below the CIE are restricted to distinct black shale layers (Tafelfleins, Seegrasschiefer) (Fig. 3). HI values ranging from 459 to 614 mgHC/gTOC in these beds reflect a high amount of amorphous aquatic organic matter in these layers (Galasso et al. 2021). Strongly anoxic conditions are suggested by the presence of low concentrations of aryl isoprenoids, high (Pr + Ph)/(*n*-C_17_ + *n*-C_18_) ratios (Fig. 7) and C_35_ HHI values (~ 0.04; Table 3), which are similar to those in the strongly anoxic CIE. However, various degrees of bioturbation described by van Acken et al. (2019), show that the redox proxies have to be interpreted with caution. Nevertheless, Pr/Ph ratios pretending dysoxic to oxic conditions (Didyk et al. 1978) are surprisingly high (2.2–2.7; see also Schouten et al. 2000; Frimmel et al. 2004). Frimmel et al. (2004) suggested that high Pr/Ph ratios reflect additional input of terrigenous pristane and/or secondary oxidation. However, both explanations appear unsatisfactory because neither an increased input of terrigenous phytoclasts (Galasso et al. 2021) nor secondary oxidation phenomena on pyrite can be observed. Very low steranes/hopanes ratios may reflect input of heterotrophic bacterial biomass involved in the degradation of algae (Peters et al. 2005).

#### Toarcian CIE (10.2–7.4m)

The presence of black shales with a distinct lamination and the widespread absence of benthic organisms is evidence for strongly oxygen-depleted conditions in the Toarcian CIE interval (Röhl and Schmid-Röhl 2005). Consequently, TOC contents increase sharply to values higher than 10 wt.% at the base of the Toarcian CIE (D67, Fleins). TOC contents remain high during the CIE and are low only in a limestone layer (“Unterer Stein”). Below and above the limestone layer, the average HI is 657 mgHC/gTOC reflecting very high amounts of aquatic organic matter as documented by macerals analysis (Fig. 4), palynofacies (palynofacies B of Galasso et al. 2021), and reduced ratios of C_19_/C_23_ and C_20_/C_23_ tricyclic terpanes.

Marine aquatic macerals include telalginite and lamalginite (Fig. 4c). Tasmanales alginite is present in all samples, but is especially abundant in sample D67 from the uppermost *tenuicostatum* Zone. Terrigenous macerals including vitrinite (Fig. 4c), recycled vitrinite, and inertinite are very rare. Fish remains (Fig. 4g) are especially abundant in samples from the *exaratum* Subzone of the *falciferum* Zone.

Pr/Ph ratios decrease gradually from 1.73 to 1.18 between 10.1 m (D67) and 8.2 m (D100) and increase to a value of 1.68 at 7.3 m (D104). The (Pr + Ph)/(*n*-C_17_ + *n*-C_18_) ratio shows the opposite trend. Following Frimmel et al. (2004), these data suggest strictly anoxic conditions with only very short intervals with oxygen availability at the sea floor during the uppermost *tenuicostatum* Zone (D67).

High concentrations of aryl isoprenoids, markers for photosynthetic sulfide-oxidizing bacteria (Summons and Powell 1987), suggest photic zone anoxia, which were previously postulated for the Dotternhausen section (Schouten et al. 2000; Frimmel et al. 2004; Schwark and Frimmel 2004). At Dotternhausen (Schwark and Frimmel 2004) and at Dormettingen, the highest degree of oxygen depletion is reached in the upper part of the negative CIE, during and after deposition of the “Unterer Stein” (Fig. 7). This level is also characterized by maxima in gammacerane index and DBT/Phen ratios supporting that oxygen depletion was linked to salinity stratification (see also Schouten et al. 2000) probably due to strong basin restriction and increased run-off (e.g., Röhl et al. 2001; Schwark and Frimmel 2004; McArthur et al. 2008). Remarkably, the most severe photic zone anoxia occurs just at the onset of the carbon isotope recovery phase (C3 after Suan et al. 2008). The abundance of methyl-alkylbenzenes is highest in samples showing the negative CIE and co-varies with the concentrations of aryl isoprenoids (Fig. 7), suggesting the formation of both compound groups through degradation of carotenoids (e.g., isorenieratene). However, in difference to Schouten et al. (2000), the presence of the isoprenoid chain could not be detected in the present study. Sinninghe-Damsté et al. (1993) and Chairi et al. (2020) reported increased contents of alkylbenzenes in sediments deposited during periods of enhanced salinity. Therefore, the formation of the methyl-alkylbenzenes, found in elevated abundances in the Toarcian CIE samples, is considered to have been accelerated in response to salinity stratification and water column anoxia. Beside degradation of carotenoids, direct cyclization and aromatization of the straight chain fatty acids are suggested as responsible mechanism (Derenne et al. 1990; Chairi et al. 2020).

Concentrations of 4-methylsteranes including dinosterane, which is generally ascribed to dinoflagellates (Withers 1987; Moldowan and Talyzina 1998; Volkman et al. 1990), are high (Fig. 7f). Galasso et al. (2021) showed that anoxic conditions resulted in a complete disappearance of dinoflagellates cysts in palynofacies B. This apparent discrepancy, which was also observed by Schouten et al. (2000), suggests the presence of specific non-cyst forming dinoflagellate species. Palynological evidence shows that dinoflagellates cysts were replaced by green algae (prasinophytes) with a high tolerance to fresh-water input and periodic photic zone anoxia and by *Spheropollenites* (Prauss et al. 1991; Galasso et al. 2021).

The sterane/hopane ratio increases within the CIE as algal biomass is better preserved. However, the sterane/hopane ratio is locally decreased in sample D100 (Fig. 7i). This is mainly attributed to high hopane concentrations, which reflect strong bacterial activity (e.g., Fonseca et al. 2021).

The 2α-methylhopane index (2-MHI) and the 3β-methylhopane index (3-MHI) have been introduced as proxies for specific bacteria. 2-MHI was used as a marker for cyanobacteria by Summons et al. (1999), but Rashby et al. (2007) added additional sources. 3-MHI was used as a marker for methanotrophs and acetic acid bacteria (Zundel and Rohmer 1985; Talbot et al. 2003; Farrimond et al. 2004). Similar to a section in the Cleveland Basin (French et al. 2014), the vertical variation of the 3-MHI is minor. However, a distinct increase of 2-MHI within the Toarcian CIE with a maximum in sample D100 above the Unterer Stein limestone marker bed (Fig. 7k) indicates enhanced activity of diazotrophic cyanobacteria at Dormettingen. Activity of diazotrophic cyanobacteria during the Toarcian CIE is also indicated by δ^15^N values near 0 recently reported from the Dotternhausen section (Wang et al. 2021) and the Rietheim section, about 90 km south of Dotternhausen/Dormettingen (Montero-Serrano et al. 2015). This is in contrast to locations in England, Wales, and Italy, where positive nitrogen isotope excursions suggest denitrification of anoxic bottom waters (Jenkyns et al. 2001).

#### Post-CIE

TOC contents remain high above the CIE and vary between 4.0 and 10.4 wt.% (Fig. 3). HI values are partly very high and values between 700 and 730 mgHC/gTOC are observed near the base of this interval and near the top of the *falciferum* Zone. Slightly lower HI values (640–680 mgHC/gTOC) occur between 7.0 and 5.2 m. This interval (below and above the “Oberer Stein” limestone bed) coincides with a few thin bioturbated layers and a moderately diverse benthic fauna (van Acken et al. 2019). Hence, the slightly lower HI values might be related to increased oxygen availability. Relatively low HI values near the top of the succession (D128, D129) might be due to weathering.

Vertical trends of Pr/Ph (1.7–1.9; Fig. 7c) and (Pr + Ph)/(*n*-C_17_ + *n*-C_18_) ratios (1.6–1.0; Fig. 7d) and the concentrations of aryl isoprenoids (89–44 µg/g EOM) (Fig. 7q) indicate that the oxygen availability increased gradually after the CIE till deposition of the lowermost part of the *bifrons* Zone (sample D118 at 4.0 m). Sample D120 (3.4 m) marks a return to strongly oxygen-depleted conditions (Pr/Ph: 1.7; (Pr + Ph)/(*n*-C_17_ + *n*-C_18_): 1.6; aryl isoprenoids: 89 µg/g EOM). Above this level, oxygen concentrations increased again. Overall, the biomarker ratios suggest anoxic to temporarily oxic conditions. The proposed redox trend fits well with the occurrence of benthic fossils around the Inoceramenbank (*falciferum/bifrons* boundary) and in the upper part of the *commune* Subzone (Röhl et al. 2001). Interestingly, the redox trend is not reflected in the C_35_ HHI (Table 3) and the DBT/Phen ratio (Fig. 7o), which suggests fairly constant availability of free H_2_S in the depositional/diagenetic environment (Hughes et al. 1995).

The increase in oxygen availability following the CIE allowed the recovery of dinoflagellate cysts, although with low numbers and diversity (palynofacies C of Galasso et al. 2021). The return of dinoflagellate cysts has been attributed to a reconnection of the German Basin to the open ocean, low numbers and diversity might be indicative of persistent adverse environment, probably due to low levels of water column mixing (Galasso et al. 2021). The latter is supported by a moderately high gammacerane index in post-CIE sediments (0.10–0.20; Fig. 7m), which suggests that the water column remained at least temporarily stratified. Despite the re-appearance of dinoflagellate cysts, concentrations of dinosteranes (and other 4-methylsteranes) are low supporting the observation that dinosteranes are probably not derived from the cyst-forming dinoflagellates.

Probably, the change in redox conditions in the lowermost *bifrons* Zone also influenced TOC and S contents, which increase in the same depth interval (4.0 to 3.7 m) from 5.2 to 9.8% and from 1.7 to 3.6%), respectively (Fig. 3). The sterane/hopane ratio is also raised at 3.4 m depth (Fig. 7i), suggesting the high productivity of eukaryotic organisms or enhanced degradation of algal organic matter.

Diasterane/sterane ratios are higher in post-CIE sediments (0.17–0.27) than in sediments deposited during the CIE (0.11–0.18; Fig. 7f). As carbonate contents are even lower in the CIE, higher diasterane/sterane ratios probably result from the less strictly anoxic conditions during deposition of post-CIE sediments (Fig. 14; Moldowan et al. 1994) rather than the clay catalytic effect (van Kaam-Peters et al. 1998).

### Compound-specific carbon isotope data

Compound-specific isotope data were measured for short-chain (*n*-C_17_ to *n*-C_19_), mid-chain (*n*-C_21_ to *n*-C_25_), and long-chain *n*-alkanes (*n*-C_27_ to *n*-C_29_). Short- and long-chain *n*-alkanes are typically attributed to marine and terrigenous organic matter, respectively (e.g., French et al. 2014). Especially interesting are isotope data for pristane and phytane, which are considered representative of marine photoautotrophic biomass (e.g., Schouten et al. 2000).

In this section, stratigraphic variations of compound-specific isotope data are investigated and discussed in relation to changes in the depositional environment. Thereafter, the compound-specific isotope record is compared to that of coeval sections in the Central European Epicontinental Basin System.

#### Stratigraphic variations of compound-specific isotope patterns

*pre-CIE *δ^13^C values of *n*-alkanes in five samples from the *tenuicostatum* Zone range from − 28 to − 33‰ (Table 4) and are characterized by an increase in δ^13^C values with increasing chain length (“positive pattern”; Fig. 13). Thus, δ^13^C values of long-chain *n*-alkanes are less negative than in any other sample. A high CPI, especially in low-TOC samples from the *tenuicostatum* Zone (D60, D62, D95), suggests that the long-chain *n*-alkanes are derived mainly from terrigenous organic matter. Therefore, it is reasonable to assume that the positive isotope pattern caused by relatively high δ^13^C values of long-chain *n*-alkanes reflects terrigenous input.

**Table 4:** Compound-specific carbon isotope ratios (δ^13^C; ‰) of Toarcian sediments from the Dormettingen section

Sample ID	Depth [m]	*n*-C_15_	*n*-C_16_	*n*-C_17_	*n*-C_18_	*n*-C_19_	*n*-C_20_	*n*-C_21_	*n*-C_22_	*n*-C_23_	*n*-C_24_	*n*-C_25_	*n*-C_26_	*n*-C_27_	*n*-C_28_	*n*-C_29_	Norpristane	Pristane	Phytane
D128	0.8	n.d		− 32.68	− 32.28	− 32.66	− 32.82	− 32.48	− 33.03	− 32.78	− 33.02	− 32.97	− 33.63	− 33.00			n.d	− 30.53	− 29.70
D127	1.9	n.d	− 32.69	− 33.12	− 32.71	− 33.43	− 33.55	− 33.87	− 34.44	− 33.85	− 34.67	− 34.01	− 34.95	− 34.81	− 33.85		− 28.36	− 31.26	− 29.81
D125	2.49	n.d	− 32.58	− 32.98	− 32.50	− 32.71	− 33.08	− 32.96	− 33.31	− 33.24	− 33.90	− 33.09	− 33.96	− 33.28			− 29.36	− 32.22	− 30.97
D124	2.6	n.d	− 33.02	− 33.48	− 33.19	− 33.43	− 33.80	− 33.63	− 34.02	− 33.91	− 33.64	− 33.07	− 33.62	− 32.74	− 33.29		− 29.02	− 32.20	− 30.35
D120	3.4	n.d	− 33.05	− 33.73	− 33.29	− 33.87	− 33.95	− 33.96	− 33.98	− 33.80	− 33.71	− 33.26	− 33.48	− 33.05	− 32.81		− 29.29	− 31.83	− 30.51
D118	4	n.d	− 32.71	− 32.93	− 32.52	− 32.97	− 33.09	− 33.03	− 33.32	− 32.66	− 32.96	− 32.43	− 32.61	− 32.77			− 29.46	− 31.56	− 30.16
D115	4.9	n.d	− 32.60	− 32.98	− 32.63	− 33.38	− 33.58	− 33.78	− 34.14	− 33.53	− 33.59	− 33.47	− 33.95	− 33.80			n.d	− 31.04	− 29.69
D112	5.5	− 31.46	− 32.20	− 32.61	− 32.30	− 32.80	− 33.02	− 33.05	− 33.39	− 32.78	− 32.86	− 32.63	− 33.28	− 33.17			n.d	− 30.62	− 29.21
D111	5.7	n.d	− 32.18	− 32.53	− 32.16	− 32.64	− 32.99	− 32.93	− 33.47	− 33.15	− 33.11	− 32.60	− 33.22	− 33.28			n.d	− 30.42	− 29.31
D109a	6	− 31.04	− 31.37	− 31.91	− 31.63	− 31.75	− 32.31	− 32.25	− 32.69	− 32.32	− 32.66	− 32.51	− 33.16	− 32.85			− 28.22	− 30.44	− 29.40
D109	6.2	n.d		− 32.76	− 32.50	− 32.75	− 33.06	− 33.06	− 33.63	− 33.49	− 33.22	− 32.84	− 33.15	− 32.73			− 28.09	− 29.92	− 29.03
D106	6.9	n.d	− 31.50	− 31.83	− 31.56	− 31.50	− 31.60	− 31.50	− 31.96	− 31.52	− 31.96	− 31.09	− 31.79	− 31.21			− 29.15	− 31.01	− 30.06
D104	7.3	− 32.10	− 32.39	− 32.96	− 32.56	− 32.97	− 33.09	− 33.25	− 33.60	− 33.02	− 33.49	− 33.26	− 33.82				− 28.63	− 30.90	− 29.59
D101	7.8	− 33.08	− 33.48	− 34.01	− 33.94	− 34.27	− 34.63	− 35.16	− 35.63	− 35.10	− 36.20	− 35.35	− 36.24				− 31.28	− 33.49	− 32.35
D100	8.2	− 31.93	− 33.08	− 34.00	− 33.79	− 33.99	− 34.56	− 34.79	− 34.97	− 34.39	− 35.52	− 35.25	− 35.87	− 35.90			− 31.79	− 34.38	− 33.19
D98	8.7	− 33.75	− 33.91	− 34.54	− 34.30	− 34.39	− 34.84	− 35.20	− 35.26	− 35.05	− 35.71	− 35.38	− 36.38	− 36.74			− 32.30	− 34.45	− 33.31
D96	9.1	− 34.43	− 34.72	− 35.20	− 35.05	− 35.30	− 35.36	− 35.65	− 36.13	− 35.81	− 36.71	− 35.48	− 36.83	− 36.73			− 33.36	− 35.27	− 34.28
D93	9.7	− 33.62	− 34.17	− 34.69	− 34.61	− 34.66	− 34.82	− 34.90	− 35.33	− 34.65	− 35.47	− 34.86	− 35.45	− 35.01			− 33.94	− 35.71	− 34.68
D67	10.1	− 33.13	− 33.35	− 33.43	− 33.22	− 33.50	− 33.55	− 33.74	− 33.83	− 33.14	− 33.73	− 32.79	− 33.11	− 33.33			− 30.26	− 32.03	− 30.87
D65	10.4	n.d		− 30.46	− 30.42	− 30.59	− 30.45	− 30.12	− 29.61	− 29.60	− 29.53	− 29.54	− 29.97	− 28.47			n.d	− 30.88	− 29.24
D62	10.7	n.d		− 31.59	− 30.83	− 30.92	− 31.46	− 31.00	− 31.35	− 30.77	− 30.37	− 29.85	− 30.50	− 29.75	− 28.38	− 28.00	n.d	− 30.11	− 31.30
D60	10.9	n.d		− 30.58	− 30.85	− 30.54	− 30.91	− 30.92	− 31.18	− 30.72	− 29.89	− 29.95	− 30.93	− 28.20			n.d	− 31.10	− 30.74
D60a	10.95	n.d	− 31.21	− 31.87	− 31.47	− 31.57	− 31.68	− 31.64	− 32.08	− 31.50	− 32.30	− 31.41	− 31.58	− 30.72			− 29.82	− 31.86	− 30.79
D57	11.3	n.d	− 31.73	− 32.57	− 32.14	− 32.15	− 31.92	− 31.67	− 32.11	− 31.44	− 32.53	− 31.54	− 31.59	− 30.69	− 31.11	− 29.82	− 30.70	− 33.31	− 32.99

δ^13^C values of pristane and phytane range typically from − 29 to − 32‰ (Fig. 15). Only the δ^13^C values of sample D57 (Tafelfleins) are characterized by δ^13^C values, which are more negative than *n*-alkanes in the same sample and more negative than pristane and phytane in any sample outside the Toarcian CIE. Similar values have been determined by Schouten et al. (2000) for their sample T6, located at the same stratigraphic level in the Dotternhausen section. This indicates that pristane and phytane are derived from aquatic organisms using very light, probably recycled carbon sources.

*Toarcian CIE *The lowermost sample (D67; Fleins) represents the uppermost *tenuicostatum* Zone and the transition from palynofacies A to B (Galasso et al. 2021). δ^13^C values of *n*-alkanes in this sample (− 33 to − 34‰) are rather uniform. Relatively high δ^13^C values of pristane and phytane (− 31 to − 32‰), which are in the order of those from the underlying rocks are another peculiarity of sample D67. The overlying samples from the main part of the Toarcian CIE (D93 to D101; lower *falciferum* Zone) represent palynofacies B. Their *n*-alkanes are isotopically light (− 33 to − 37‰; Figs. 12, 13) and δ^13^C values decrease significantly with increasing chain length (negative trend; Fig. 13). Isoprenoids of these samples are also isotopically light (− 31 to − 36‰), but δ^13^C values in some of these samples are distinctly less negative than those from *n*-alkanes (Figs. 12, 13).

The uppermost sample (D104; middle *falciferum* Zone) has been attributed by Galasso et al. (2021) to palynofacies C, but still contains very little terrigenous organic matter. It includes *n*-alkanes with a similar distribution of δ^13^C values like sample D67, but δ^13^C values of isoprenoids are less negative (Fig. 13).

Stratigraphic trends of δ^13^C values of *n*-alkanes and isoprenoids (pristane, phytane) across the CIE show remarkable differences (Fig. 12). Short- and mid-chain *n*-alkanes show comparable trends within minimum δ^13^C values in sample D96 (9.1 m). The record for long-chain *n*-alkanes is incomplete because of their low abundance in the CIE, but δ^13^C values of *n*-C_27_ reach minima (-36.7‰) in samples D96 and D98 (8.7 m) producing an overall symmetric depth trend across the CIE. In contrast, δ^13^C values from pristane and phytane show a clear asymmetric trend. δ^13^C values in sample D67 (Fleins) are still relatively high and similar to that of pre-CIE sediments. The minimum values are reached already at a depth of 9.7 m in sample D93 (Pr: − 35.7‰; Ph: − 34.7‰). Following the subdivision of the CIE by Suan et al. (2008), this sample coincides with the end of phase C1. Above this level, δ^13^C values increase slowly during phase C2 (sample D100; 8.2 m; Pr: 34.4‰; Ph 33.2‰) and more rapidly during phase C3 (sample D104; 7.3 m; Pr: − 30.9‰; Ph: − 29.6‰).

The data show that there is an obvious time lag (~ 450 kyr according to the calibration of Suan et al. 2008) between the minimum of δ^13^C_CO2_ in the photic zone as reflected by the δ^13^C_isoprenoid_ minimum and the maximum of oxygen depletion, related to the shallowest position of the chemocline, represented by sample D100 (Fig. 7). This suggests that there might be an external source for isotopically light CO_2_ (e.g., dissociation of methane hydrate; e.g., Hesselbo et al. 2000a, b; Kemp et al. 2005). Apart from that, recycling of isotopically light carbon from deeper levels of the stratified water body (“Küspert model”; e.g., Küspert 1982; Jenkyns 1985; van de Schootbrugge et al. 2005) certainly also contributed to the light isotopy, especially during the time of maximum oxygen-restriction.

The amplitude of the CIE varies significantly for different compounds (Fig. 12). It is about 4.5‰ for short-chain *n*-alkanes, about 5–6‰ for mid-chain *n*-alkanes, and up to 9‰ for *n*-C_27_. Pristane and phytane show a negative shift of ~ 5‰. A slightly higher shift (~ 6‰) is found for bulk organic carbon. As discussed in a following section, the very high negative shift for *n*-C_27_ may be related to the combined effect of atmospheric CO_2_ levels and climatic factors (e.g., Lomax et al. 2019; Ruebsam et al. 2020) or to the described change from terrestrial (pre-CIE) to marine organic matter input (CIE; see also Suan et al. 2015). These mechanisms are probably not valid for short-chain *n*-alkanes, pristane, and phytane. Therefore, an amplitude of 4.5 to 5.0‰ seems realistic for the Toarcian CIE at Dormettingen. This value is slightly higher than the one proposed by Suan et al. (2015) for Dotternhausen, based on correlations between HI and δ^13^C values (3–4‰).

*Post-CIE *Sediments deposited after the Toarcian CIE contain *n*-alkanes with δ^13^C values between − 31 and 34‰. These values either remain constant with increasing chain length (e.g., D106, D120) or decrease slightly (e.g., D109a). δ^13^C values of isoprenoids are less negative (− 28 to − 32‰; Fig. 13).

*n*-Alkanes with varying chain lengths exhibit similar vertical trends (Fig. 12). δ^13^C values reach a maximum in sample D106 (6.9 m) near the top of the CIE and decrease upwards to sample D127 (1.9 m). The uppermost sample (D128, 0.8 m) has less negative values than the underlying sample D127. A shift towards less negative δ^13^C values in mid- and long-chain *n*-alkanes is observed between samples D115 (4.9 m) and D118 (4.0 m), which coincides with the boundary between palynofacies C and D according to Galasso et al. (2021). Therefore, the isotope record may reflect slightly enhanced input of terrigenous organic matter.

Pristane and phytane show a different trend. Their δ^13^C maximum is observed at 6.2 m (sample D109) and there is a remarkably uniform decrease in δ^13^C values to sample D125 at 2.5 m (Fig. 12). Apart from the change of palynofacies C to D, additional environmental changes occurred during deposition of the post-CIE sediments. These include the decrease in oxygen availability between samples D118 and D120 (4.0–3.4 m). Moreover, Mattioli et al. (2009) showed a strong increase in nannofossil flux within the lower part of the *falciferum* Subzone in the Dotternhausen section. At Dormettingen, this position corresponds to a depth of ~ 5.9 m (sample D110). Apparently, none of these events had a significant effect on isotope ratios of pristane and phytane (Fig. 12). There is an excellent agreement between the isotope data of isoprenoids measured by Schouten et al. (2000) and the new data. However, data from a sample, which falls into the gap between sample D127 and D128 (their sample T45) is isotopically lighter than these samples. This may indicate that δ^13^C values in the uppermost part of the section might be more strongly varying than indicated by our data. Overall, the data suggest that the carbon pool in the photic zone following the Toarcian CIE changed more gradually than indicated by the strongly varying δ^13^C values of bulk organic matter.

#### Comparison with compound-specific carbon isotope records from other basins

Apart from the pioneering work of Schouten et al. (2000), compound-specific isotope records across the Toarcian CIE are rare. δ^13^C values have been determined for phytane and isorenieratane in the Paris Basin (ANDRA Core HTM 102) (van Breugel et al. 2006). The most detailed record has been provided from the Hawsker Bottoms section (Cleveland Basin) by French et al. (2014) (see also Sælen et al. 2000). Data for long-chain *n*-alkanes in the Sichuan Basin (China) and the Iberian Basin (La Cerradura; Spain) were published by Xu et al. (2017) and Ruebsam et al. (2020).

The comparison of the Dormettingen section with those in the Cleveland, Paris, Iberian, and Sichuan basins shows major differences in the amount of the negative CIE:

The negative CIE of bulk organic matter at Dormettingen (6–7‰) and in the Cleveland Basin (~ 5–7‰) is similar, whereas the negative CIE in the sections in the Paris (3.7‰), Iberian (~ 3‰) and Sichuan basins (~ 4%) is smaller.

Short-chain *n*-alkanes (*n*-C_17_, *n*-C_18_, *n*-C_19_) and the isoprenoids pristane and phytane are considered as derived from marine organisms. They display relatively small negative CIEs of only ~ 2–3 (*n*-alkanes) and ~ 2‰ (isoprenoids) at Hawsker Bottoms and larger CIEs of ~ 4.5‰ and ~ 5‰ at Dormettingen. The higher CIE magnitude results in isotopically lighter pristane and phytane at Dormettingen (Pr: − 35.7‰; Ph: − 34.7‰) compared to those at Hawsker Bottoms (Pr: − 33.0‰; Ph: − 33.9‰). Probably the higher CIE magnitude reflects more severe restriction in the SWGB. Another difference between the isoprenoid records at Hawsker Bottoms and Dormettingen concerns the vertical trend of pristane and phytane δ^13^C values. At Hawsker Bottoms, δ^13^C values decrease sharply already in the middle part of the *semicelatum* Subzone and remain constant across the *tenuicostatum/falciferum* boundary and within the entire CIE. At Dormettingen, the main shift is at the *tenuicostatum/falciferum* boundary, and δ^13^C increases upwards within the main part of the CIE. A CIE with intermediate magnitude has been observed in the phytane record (3.3‰) in the Paris Basin (van Breugel et al. 2006).

At the latter locality, the relative abundance of isorenieratane increased after the δ^13^C of phytane and bulk organic matter return to more positive values (van Breugel et al. 2006). This suggests that the temporal decoupling of the maximum isotope excursion and the peak in oxygen depletion is not restricted to the SWGB. In the Cleveland Basin, the time shift of the maximum oxygen depletion and maximum isotope excursion is less obvious, as δ^13^C values of pristane and phytane remained constant during the entire CIE.

Long-chain n-alkanes (*n*-C_27_, *n*-C_28_, *n*-C_29_) are typically derived from terrigenous organic matter. In pre-CIE sediments their δ^13^C values are similar at Dormettingen (− 28.2 to − 31.1‰; Table 4) and in the Cleveland Basin (− 27.8 to − 30.0; French et al 2014), but significantly higher in the Iberian Basin (− 23.8 to − 25.5; Ruebsam et al. 2020). Differences in absolute δ^13^C values of terrigenous *n*-alkanes in the Cleveland Basin, located in a winter-wet temperate climate belt, and the Iberian Basin, located in a winter-wet to semi-arid climate belt (Rees et al. 2000), have been related to different precipitation rates and floral assemblages (Ruebsam et al. 2020). Thus, a winter-wet temperate climate during the *tenuicostatum* Zone at Dormettingen is in agreement with the isotope data.

Long-chain *n*-alkanes in pre-CIE sediments show a much larger negative CIE of ~ 8–9‰ in Dormettingen (Fig. 12) than in the Cleveland Basin (~ 4–5‰; French et al. 2014). Even smaller shifts (~ 3.7‰) are reported for the sections in the Iberian Basin and lacustrine rocks in the Sichuan Basin (Ruebsam et al. 2020; Xu et al. 2021). Interestingly the vertical trends in these basins are very different and show rather uniform values (Cleveland Basin), two minima (Iberian Basin), or a slow decrease followed by a rapid increase (Sichuan Basin). Assuming that the amount of CO_2_ in the atmosphere and its δ^13^C value changed uniformly worldwide, the observed differences may reflect regional climate (e.g., precipitation) variations (e.g., Schubert and Jahren 2013; Lomax et al 2019). In addition, the extraordinarily high shift at Dormettingen may be influenced by a major change in organic matter input (see also Suan et al. 2015). Whereas a terrigenous source of the long-chain *n*-alkanes in the pre-CIE rocks is proven based on biomarker and palynological evidence, *n*-C_27_ in the Toarcian CIE may have an algal origin.

## Conclusion

**Fig. 16:**
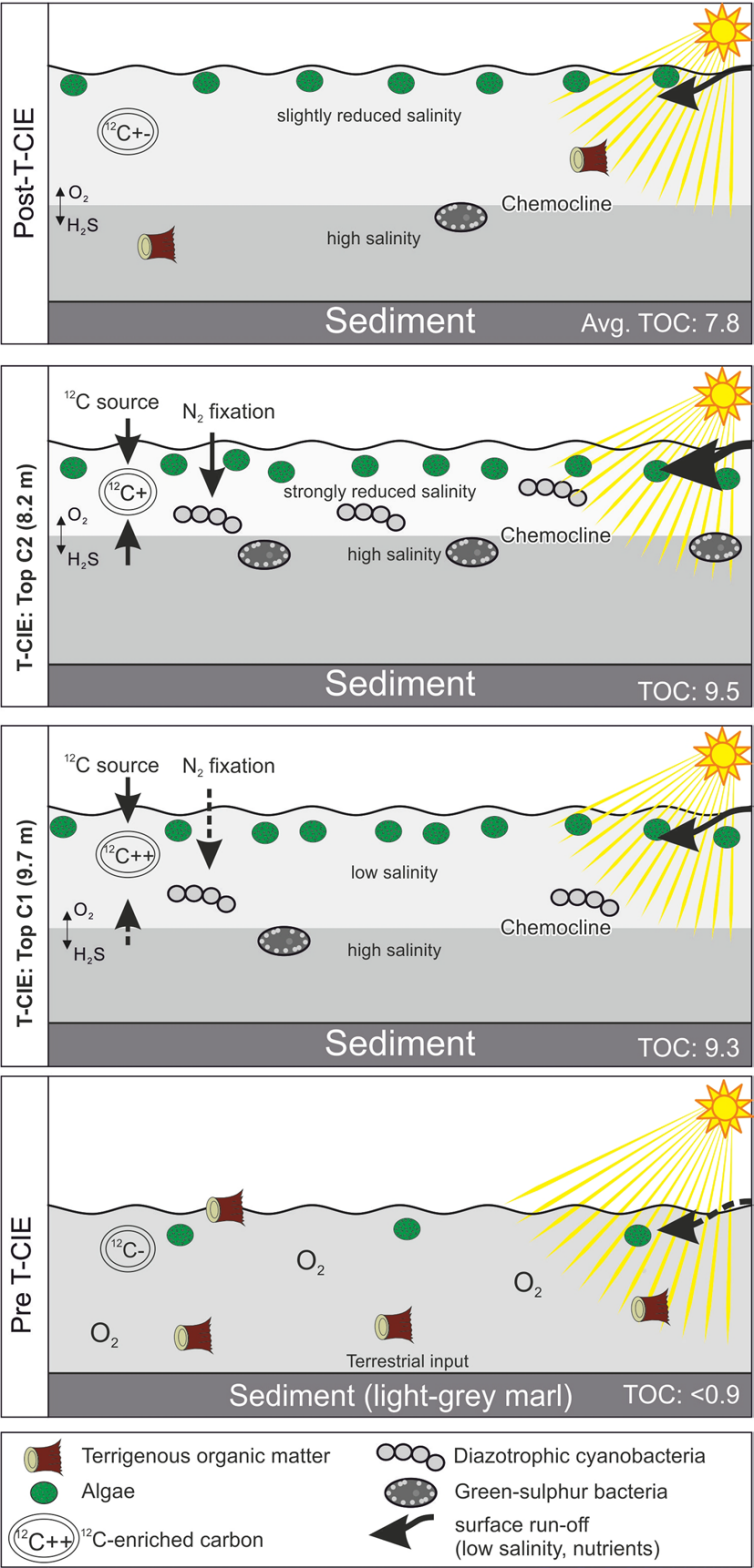
Cartoon showing amount and type of organic matter input, changes in salinity and the position of the chemocline, as well as the degree of surface run-off, nitrogen fixation by cyanobacteria, and the source of isotopically light CO_2_. Photic zone anoxia allowed the life of green sulfur bacteria

The paper presents a comprehensive organic geochemical study of the Dormettingen section (SW Germany). It expands the knowledge of the Toarcian evolution in the Southwest German Basin and provides new insights into the factors controlling carbon isotopy on a molecular level. The most important results are summarized below and displayed in a cartoon (Fig. 16).

The evolution of the depositional environment at Dormettingen was similar to that in nearby Dotternhausen. In both sections, photic zone anoxia, indicated by a variety of different biomarkers, prevailed during the Toarcian CIE. As postulated by previous authors, anoxia was caused by basin restriction and a stratified water column. The chemocline reached its shallowest position above the “Unterer Stein” marker horizon (sample D100; 8.2 m).

Elevated 2α-methylhopane index values in this interval reflect the enhanced activity of diazotrophic cyanobacteria. This interpretation is strongly supported by nitrogen isotope values at Dotternhausen (Wang et al. 2021). Obviously, denitrification, which controlled nitrogen isotopy in other basins during the Toarcian CIE, played a subordinate role emphasizing differences in geological settings (basin restriction versus upwelling).

Pristane and phytane are derived from photoautotrophic marine biomass. Their δ^13^C values show a very strong negative excursion (~ 5‰) and are partly very low (minimum: − 35.7‰) in the lower part of the Toarcian CIE (sample D93; 9.7 m). Above this point, an increase in δ^13^C values of pristane and phytane is observed. Hence, there is an obvious time shift of about 450 kyr between the isotopic minimum and peak anoxic conditions. This suggests that recycled isotopically light dissolved inorganic carbon from deeper levels of the stratified water body (“Küspert model”) is probably not the only source for isotopically light carbon.

*n*-C_27_ displays the largest negative CIE (~ 9‰). This very high magnitude is due to the combined effect of the global CIE and a major change in the source of the organic matter. While *n*-C_27_ in pre-CIE sediments is mainly derived from terrigenous organic matter, *n*-C_27_ within the CIE is derived from marine organic matter.

Despite that, the actual magnitude of the CIE, as reflected by short-chain *n*-alkanes (~ 4.5‰) and isoprenoids, is higher in the Southwest German Basin (Dormettingen) than in other subbasins of the Central European Epicontinental Basin.

## Data Availability

Data in the manuscript and appendix will be published as replication data set in Pangaea.
